# Trust then talk or talk then trust? The coevolution of communication networks and inter-organizational trust

**DOI:** 10.1186/s41235-025-00662-1

**Published:** 2025-09-19

**Authors:** Sean M. Fitzhugh, Cynthia K. Maupin, Arwen H. DeCostanza

**Affiliations:** 1 Humans in Complex Systems Division, DEVCOM Army Research Laboratory, Aberdeen Proving Ground, MD 21005 USA; 2 Department of Management, University of Mississippi, University, MS 38677 USA; 3 Technology Transition Office, DEVCOM Army Research Laboratory, Aberdeen Proving Ground, MD 21005 USA

**Keywords:** Trust, Networks, Communication, Group dynamics, Organizational behavior

## Abstract

Trust serves an important purpose in organizations composed of numerous, specialized, interdependent roles. Supporting confidence that individuals will dutifully fulfill the responsibilities of those roles without causing harm to the organization, trust enables coordinated task execution across multiple roles and facilitates information exchange among individuals by reducing cognitive resources spent verifying information accuracy and reliability. Interactions play an important role in shaping and updating trust, but the mechanisms underlying the relationship between communication networks and trust dynamics remain poorly understood. This paper addresses that gap by directly examining the coevolution of communication networks and trust. During a multi-day military training exercise, participants (n=83) from three distinct units formed a coalition organization largely focused on collecting, analyzing, and acting on information gleaned from the operating environment of roughly 10k units under their command. Over the course of the exercise, each participant provided eight ratings of trust in their own unit and their coalition partners’ units. Static and dynamic network models of the organization’s communication networks assessed whether trust is an antecedent or product of communication. Results consistently show that when individuals report elevated trust in a unit, they become more likely to form and sustain relationships to members of that unit during the next time period. They also increase their rates of communication to those unit members. However, this relationship does not work in reverse: Increased communication to a unit does not precede increased trust in that unit. These findings suggest temporal directionality in the coevolution of trust and communication.

## Introduction

Trust serves an essential role in sustaining organizational functionality (Kramer & Tyler, 1996). Trust is commonly defined as “the willingness of a party to be vulnerable to the actions of another party based on the expectation that the other will perform a particular action important to the trustor, irrespective of the ability to monitor or control that other party” (Mayer et al., 1995, p. 1171). Establishing these essential expectations has important implications for organizations composed of specialized, interdependent roles. Supporting broader organizational goals, individuals in these roles strive to achieve coordinated task execution (Galbraith, 1977). As an organizing principle, trust allows these specialized roles to exist without engendering suspicion that individuals will take advantage of those positions, such as loafing while expecting others to pick up the slack or taking undue benefits (McEvily et al., 2003). Trust in fellow organizational units (individuals, teams, departments, etc.) enables coordination as individuals prioritize larger, organizational-level goals over their own individual- or team-level goals (DeChurch et al., 2012). Trust serves an important function in organizations—it shapes a wide variety of group processes such as cooperation (Boyle & Bonacich, 1970; Kramer, 1999; Muthusamy & White, 2005; Serva et al., 2005; Von Lampe & Ole Johansen, 2004), monitoring (Langfred, 2004), and conflict management (Zaheer et al., 1998; Simons & Peterson, 2000), and outcomes such as performance (Zaheer et al., 1998; Langfred, 2004; Akgün et al., 2015). Recognizing the importance of trust in group settings, scholars of organizational, team-based, and group research have devoted considerable attention to the topic in recent decades (Costa, 2003; Mayer et al., 1995; Rousseau et al., 1998).

In addition to preserving organizational functionality driven by interdependence, trust plays an important role for managing individual cognition within organizations. Information exchange is often predicated on formation and sustainment of interpersonal relationships, and trust serves as a heuristic to conserve cognitive effort that would otherwise be used to verify the accuracy and quality of information from that partner (McEvily et al., 2003). Additionally, relationships endowed with trust often lack monitoring to detect opportunistic behavior, thus further preserving cognitive resources (Uzzi, 1997). Trust therefore catalyzes information exchange between parties. Further shaping the role of trust on cognitive demands, the forging and preservation of relationships require effort (Cummings et al., 2006), and the costs of searching for and establishing new partnerships may lead individuals to rely on a stable set of trusted partners rather than continuously expending cognitive effort searching for optimal partners (Granovetter, 1985; Mariotti & Delbridge, 2012). That is, relying on trusted partners often has lower cognitive demands even if potentially more valuable but yet-untrusted partners may exist (Mariotti & Delbridge, 2012). Indeed, failing to manage the burden of sustaining communication partnerships leads to overload and a need to prune one’s communication network (Elfring & Hulsink, 2007; Oldroyd & Morris, 2012). Trust therefore not only reduces the cognitive resources expended on existing information exchange relationships, but it encourages individuals to avoid the burden of forming and sustaining new relationships in response to changing information requirements.

Trust has been conceptualized in a variety of ways, depending on the context and referents for which the trust relationship exists (Castaldo et al., 2010). This paper’s intra-organizational context lends itself to focusing on competence-based trust (Butler Jr & Cantrell, 1984), where the level of trust assigned to a trustee is a function of confidence in the trustee’s ability to advance organizational goals. With this conceptualization, the vulnerability inherent to trust-based relationships lies in the risk that the organization will be unable to achieve its goals if the trustee’s obligations go unfulfilled. Trust has unique implications and outcomes for a relationship depending on whether the referent (i.e., the trustee) is an individual, a group (e.g., a collective unit within an organization), or an abstract entity (e.g., institutions that serve as the foundation for a democracy) (Fulmer & Gelfand, 2012). In this paper, we focus on instances in which the referent of trust is an organizational sub-group; this type of trust that has received considerably less attention than trust between individuals (Robinson, 1996; Kramer, 1999; Fulmer & Gelfand, 2012). Although the trust literature has recognized the importance of trustor–trustee interactions on trust (Rousseau et al., 1998), scholarly understanding of the interrelationship between organizational trust and communication network characteristics remains understudied (Fulmer & Gelfand, 2012). Moreover, due to the sparsity of cross-sectional investigations, temporal ordering regarding how trust impacts communication patterns and vice versa remains largely unexplored (De Jong et al., 2016).

In this paper, we address these gaps by assessing trust dynamics within an organization while incorporating both network and temporal perspectives. Specifically, we leverage a relational, network-based perspective to represent dynamically changing trust relationships that exist between organizational units in the presence of changing task demands. Utilizing repeated measures of individuals’ trust in organizational units within a large, multifaceted military organization over the course of a training exercise, we extend theories largely borne from static conceptualizations of trust to dynamic measures of trust in order to identify where past theory explains trust dynamics and where theoretical gaps remain. We begin by highlighting the interrelation among trust, communication networks, and temporal dynamics before utilizing theories of trust to develop hypotheses explaining how trust shapes decisions to form relationships and the rate of information flow across those relationships. We then describe our organizational data and clarify how static and dynamic models of organizational communication networks will allow us to probe trust dynamics. Lastly, we review an extensive set of results and reflect on how this shapes our understanding of trust and highlights opportunities to identify new directions in trust research.

## Trust, communication networks, and time

Trust is inextricably linked to communication networks and temporal dynamics, yet these aspects are frequently overlooked in the extant literature (Fulmer & Gelfand, 2012). Trust belongs to the category of emergent group states, defined as “the behavioral interaction that occurs among members of the team enabling them to integrate their task activities toward the attainment of a group goal” (Leenders et al., 2016, p. 93). These states—such as shared mental models, collective efficacy, potency, and situation awareness—emerge and evolve as a function of interactions between individuals. Thus, repeated interactions provide a foundation for individuals to establish trust in one another and continuously re-evaluate whether that trust is justified (Bromiley & Cummings, 1995). Accordingly, trust tends to vary as a function of parties’ interactions over time (Mayer et al., 1995; Kramer, 1999). By capturing the structure and timing of those interactions, analysis of communication networks provides insight into how trust evolves. The decision-making processes underlying network dynamics—which relationships individuals form, the rates of exchange across relationships, and which relationships they decide to dissolve—provide key insights into the coevolution of trust and communication. Capturing structural and individual-level features of interaction, network analysis allows the disentangling of trust-driven features of the network from factors at the individual level (attributes such as knowledge, experience, and role), the relationship level (familiarity and prior utilization), and the structural level (reciprocity and centrality). This helps to isolate the relationship between trust and individuals’ decisions about which relationships to develop and utilize, net of other factors shaping which relationships they utilize. As a function of both past relations and expectations of future behavior, trust is an inherently temporal state, although the network implications for trust remain unclear (Zaheer & Harris, 2006). While interaction is an essential element of trust and other emergent group states, little is known about how temporal factors shape those states, particularly outside laboratory environments (Zaheer et al., 1999; Marks et al., 2001; Ilgen et al., 2005; Leenders et al., 2016; Pilny et al., 2016).

Early studies of trust leveraging a temporal perspective found that temporal ordering plays an important role in shaping the trajectory of trust. For example, in a series of Prisoner’s Dilemma games individuals were more likely to continue cooperating with a “reformed sinner,” someone who began consistently cooperating after a history of defections, than they were to cooperate with a “lapsed saint,” someone who had begun consistently defecting following a history of cooperating (Harford & Solomon, 1967). Boyle and Bonacich (1970) utilize these results to emphasize the importance of capturing dynamics in trust by demonstrating path dependency in trust relations. Despite these initial investigations into trust dynamics, subsequent studies of the mechanisms of trust dynamics have since failed to emerge. The importance of time in shaping trust relations implores us to utilize dynamic analyses of networks to characterize the relationship between communication networks and trust. This dynamic approach allows us to determine whether individual decisions about which partners to interact with reflect prior trust or shape subsequent trust. A dynamic network approach is essential because aggregation of dynamic interactions into static networks obscures temporal ordering (Quintane et al., 2014; Schecter & Contractor, 2017), often obscuring path dependence in evolution of individual and group states. It is therefore imperative to capture network dynamics when measuring trust because cross-sectional network analyses often render temporally directed relations ambiguous (Brass et al., 2004).

Beyond trust, dynamics remain poorly understood in groups, particularly for emergent states that arise based on interactions (Cronin et al., 2011). Developing a time-resolved understanding of organizational phenomena is necessary to elucidate time-dependent processes. We currently know little about time lags, feedback loops, path dependence, and durations of individual and group states, which obscures our understanding of when and for how long to measure key variables that shape those states (Leenders et al., 2016; Carter et al., 2018). By accounting for how local interaction dynamics shape group dynamics, which then continue to shape local interaction dynamics, the field will be better able to extend existing theories or develop new theories of group behavior.

The increasing availability of near-continuous logs of human behavior offers a solution to the longstanding challenge of understanding the relation between time and group behavior (Butts, 2008; Pilny et al., 2016; Schecter & Contractor, 2016, 2017; Mathieu et al., 2017). Data collected continuously through sensors or telecommunications networks provide access to discrete sequences of interaction data such as call logs, message logs, email chains, and face-to-face communication. Especially when coupled with high-resolution attribute data from individuals, ”leveraging such continuous steams of data is the key to unlocking the survey and human observation shackles limiting progress in teams’ research” (Mathieu et al., 2017, p. 461). These types of rich interaction dynamics offer a fruitful avenue for better understanding trust dynamics (Serva et al., 2005) and help to satisfy the longstanding demand for a richer understanding of how trust varies over time in organizational contexts (Lewicki et al., 2006; Serva et al., 2005; Zaheer et al., 1998). In this paper, we utilize a rich log of interaction data coupled with frequent measures of trust to characterize the relationship between the two.

## Trust dynamics

The primary goal of this paper is to characterize trust in relation to the decisions individuals make about which communication partners to utilize across a multifaceted organization. We therefore test hypotheses evaluating the association between trust and *which* relationships are utilized and the association between trust and *how often* those relationships are utilized. Motivating theory for our first set of hypotheses largely arises from theories of relational forms of exchange. One such form of relational exchange comes from social exchange theory (Gouldner, 1960; Blau, 1964; Cropanzano & Mitchell, 2005), which asserts that repeated transactions will endow the relationship with attributes, such as trust, that transcend the transactional nature of interactions (Molm et al., 2000). Informal norms emerge to govern the exchange relationship (Podolny & Page, 1998; Zaheer & Harris, 2006). By establishing feelings of personal indebtedness and reliability, these informal norms may obviate the need for expensive, formalized mechanisms of administration or oversight such as contracts (Powell, 1990; Rousseau et al., 1998). This reduces the cognitive burden of sustaining and utilizing those relationships and facilitates their prolonged existence. By reducing risk of participating in the exchange relationship, these norms facilitate the exchange of goods, services, knowledge, or information in intra-organizational contexts.

While trust catalyzes exchange, it remains unclear whether increased trust precedes increased exchange or vice versa, particularly in organizational contexts. Empirical studies demonstrate that longstanding relations reduce perceptions of opportunistic behavior and increase trust within the relationship (Parkhe, 1993; Bromiley & Cummings, 1995; Zaheer et al., 1998; McEvily et al., 2003). Similar findings emerge when the referent of trust is an organization rather than a single person (Gulati, 1995). Trust facilitates intra-organizational relationships due to enhanced interpersonal commitment and lowered costs for information exchange (Cropanzano & Mitchell, 2005), but the interrelations among trust, interaction, and time remain unclear (Zaheer & Harris, 2006; Marks et al., 2001; Leenders et al., 2016). That is, a gap exists in understanding the temporal directionality of individual decisions about which relationships to utilize. Does increased trust precede greater likelihood of communication or does communication shape trust? We posit two competing hypotheses to examine how time lags may play a role in the relationship between communication and trust. The former expects higher trust to forecast greater likelihood of communication while the latter expects greater likelihood of communication to forecast elevated trust.

1. When individuals report higher trust in a group, they will subsequently be more likely to have communication ties to members of that group.
2. When individuals have more communication ties to members of a group, they will subsequently be more likely to report higher trust in that group.

Moving beyond analysis of the presence of relationships, our next set of hypotheses examines *rates* of interaction across trusting relationships. Many relationships characterized by elevated trust establish said trust via an extended series of interactions. Building trust therefore entails the costs associated with engaging in those repeated interactions. Searching for, establishing, and maintaining relationships are costly processes and individuals have finite capacity to engage in such activities (Ahuja, 2000; Boorman, 1975; Whittaker et al., 2002; Krackhardt, 1994). Accordingly, we tend not to see a proliferation of these high-cost, high-trust relationships, and frequently individuals will decide to sustain a relationship with a good-but-not-ideal partner due to the potential costs of trying to find a more ideal partner (Granovetter, 1985).

Theories of trust rooted in bounded rationality conceptualize trust as a mechanism to offload the cognitive burden of repeated follow-up and interaction. Trust may serve as a heuristic to conserve cognitive effort that would otherwise be expended to verify the accuracy and quality of products (e.g., goods, information, services) received from the referent of trust (McEvily et al., 2003; Uzzi, 1997). According to this line of reasoning, trust would be unnecessary if everyone had perfect information because individuals could reliably forecast all actions of their partners. Lacking such all-knowing capabilities, individuals instead may rely on low levels of oversight in trusting relations and higher oversight in low-trust relations. Conducting interviews across a wide variety of organizations (Abrams et al., 2003) repeatedly spoke with managers who reported elevated levels of checking and double-checking what was said by a partner in a low-trust relationship. Even for conversations about trivial matters individuals reported exerting considerable effort to decode truth and subtle meaning in statements from low-trust alters. In groups with higher trust, however, shared terminology, understanding, and expectations aided the efficiency and comprehension of communication. As a result, individuals decided to sustain lower communication rates with those they trusted and higher communication rates with those in whom they had little trust. Rather than effective exchange of information between high-trust partners, exchange among lowly trusted individuals became bogged down with “unproductive or meaningless babbling” (Sarker et al., 2011, p. 299).

In addition to the time and effort spent on communication, individuals in low-trust relationships often engage in monitoring (e.g., surveillance, regular check-ins with superiors), which carries social, logistical, and cognitive costs (Langfred, 2004; Von Lampe & Ole Johansen, 2004). This monitoring may signal to members of the group that they are untrustworthy, which further dampens trust within the group. This aligns with results from Langfred (2004), who found a negative relationship between monitoring and trust, as high-trust groups of four tended to be less likely to engage in monitoring behaviors than their counterparts with lower trust. With less stringent requirements for detailed process information and monitoring (e.g., extended progress reports), higher levels of trust can lower volumes of communication within groups (Muthusamy & White, 2005). This is closely related to the concept of heedful interrelating (Weick & Roberts, 1993), in which a shared understanding of group responsibilities obviates the need for extensive information exchange during task completion. This reduces the burden on an individual in a high-trust relationship and catalyzes information exchange, when necessary. This array of findings in the contexts of economic and information exchange suggests that elevated communication levels may indicate monitoring or information-decoding, suggesting a lack of trust.

While some findings suggest that higher-trust relationships need not be burdened by excessive communication, others suggest that such relationships are characterized by *increased* information exchange. This underscores a larger issue of conflicting findings in the literature that obscures our understanding of how inter-organizational trust operates (Zaheer & Harris, 2006). Abrams et al. (2003) reflect these mixed findings. Despite interviews suggesting increased communication characterized by verification and double-checking in low-trust partnerships, they also found that higher-trust pairs engaged in elevated levels of productive dialogue and discussion when trying to solve problems. The latter conflicts with the notion of heedful interrelating, which characterizes groups with higher-trust levels. Within and across organizations, enhanced trust enables easier exchange of goods that lack a clear, tangible value, such as specialized and proprietary knowledge (Uzzi, 1997). Reciprocity and reliability implied by strong, trusting relationships help to ensure that when one organization provides such goods to another, that favor will be returned in the future. These mutual obligations catalyze knowledge transfer across organizations (Muthusamy & White, 2005; Zaheer & Harris, 2006).

In problem-solving contexts, enhanced trust allows individuals to form a buffer between task conflict and relationship conflict (Simons & Peterson, 2000). Lacking concern that disagreements about task-related beliefs and ideas will be misinterpreted as interpersonal hostility, individuals become more comfortable airing disagreements with those they trust (Murnighan & Conlon, 1991). This trust-induced buffer leads to increased information exchange and may contribute to improved group performance (Jehn, 1995). Owing to lower barriers for exchange of specialized knowledge and more extensive discussions during problem solving, high-trust relationships may be characterized by elevated volumes of communication. From the preceding paragraphs, we develop two competing hypotheses about how trust mediates communication flows across relationships.

Hypothesis 2a: When individuals have stronger trust in a group they will have larger volumes of communication to members of that group
Hypothesis 2b: When individuals have lower trust in a group they will have larger volumes of communication to members of that group

## Data

This paper focuses on inter-organizational trust and communication among members of a large, multifaceted military organization during a training exercise. Twenty-one members of a US Brigade staff and 16 members of a UK Brigade staff operated under the command of 46 members of a US Division staff, although neither Brigade was organic to that Division. As such, the larger organization represents the temporary merger of three groups for the duration of the exercise. These types of organizations frequently integrate into coalition partners’ operations, and this exercise provided a valuable opportunity to observe and understand information exchange and operations in these contexts. Typically responsible for 3,000 to 5,000 Soldiers, Brigade staffs have broad and expansive responsibilities. Overseeing multiple Brigades, Division staffs have even more comprehensive responsibilities. As such, much of their activity involves gleaning information from their units’ areas of operations, disseminating information, developing plans based on that information, and distributing plans and orders down the hierarchy. Requiring extensive coordination across a series of interdependent roles in order to achieve organizational goals, this organization’s behavior ought to generalize to other organizations composed of specialized, interdependent roles.

In addition to their Brigade and Division sub-groups, members of this organization fulfilled a series of interdependent, differentiated roles. Organizational members belonged to one of nine functional cells, each focused on a specific domain of operations. For example, the intelligence cell requests and analyzes information from a variety of sources to help the organization better understand its terrain, weather, civil considerations, and potential adversaries in the area of operations. Another example, movement and maneuver mobilize personnel and equipment as necessary to support the organization’s ongoing tasks. Other cells include sustainment, liaison, protection, signal, and civil affairs. Specialized roles populate each of these functional cells and these roles work interdependently within and across cells to enable the organization to achieve coordinated task execution.

Operating in a large, urban environment with a population of about 550,000, the organization had two primary goals over the course of the training exercise: disrupt operations of a local terrorist group that had been trying to portray the local government as illegitimate and ensure that an upcoming election takes place without disruption or undue influence. Supporting these goals required completing a wide variety of tasks, such as assessing local levels of anti-government and anti-coalition sentiment and disrupting distribution of illicit weapons and explosive-making materials. Over the course of the exercise the organization monitored and responded to hundreds of events in their area of operations, scheduled and planned in advance by staff administering the training (although unknown in advance to the organization undergoing training). These included mundane tasks such as infrastructure repair, media interviews, and distribution of aid as well as sudden, urgent events such as responding to mass casualty events, recovering incapacitated vehicles, and fixing telecommunications blackouts. Over the course of the training exercise, the organization completed an extremely broad range of tasks.

Due to the size and scope of their ground units and area of operations, the organization utilized a simulated operational environment. Participants sat in an array of control rooms where each used a computer workstation to send and receive information and to communicate with fellow participants, an environment similar to their typical setting during real operations. Additionally, they utilized information feeds and maps projected on the walls of their rooms; these provided information about their ground units’ current locations and activities. These several thousand ground units were simulated actors in a virtual world, however. In a program called OneSaf, virtual actors received and responded to orders issued by members of the organization, as they would during real operations. However, tactical outcomes within the training exercise were decided by this simulation program based on participants’ actions and orders. To ensure reasonably smooth operation, participants spent a week prior to the training exercise familiarizing themselves with their control and information systems and their roles and functions within the organization.

### Email communication networks

Participants primarily relied on electronic communications and we captured a complete log of email communication over the course of the exercise. Logging information on the sender, receiver, and timestamp, this email communication log captures interactions within the intra-organizational communication network. Although individuals relied to a limited extent on face-to-face and telephone communication, they stated in post-exercise surveys about communication media that they preferred email communication due to the spatial constraints on their environment. Additionally, they rated email communication as their most important communication medium and rated it highly as a source for gathering information, disseminating instructions, and receiving urgent communication. Further emphasizing the importance of email, orders in these settings are always issued in writing. Even verbal orders issued during high tempo operations are always followed by written orders. Over the course of this exercise participants sent over 6,000 emails.

**Fig. 1:**
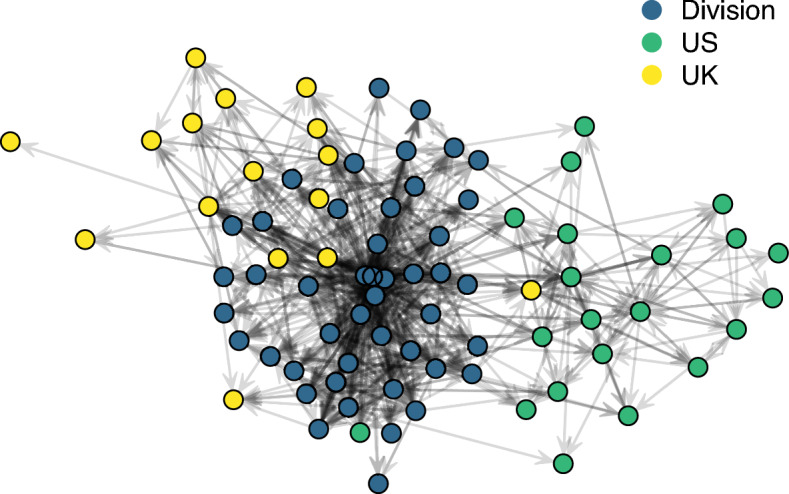
A time-aggregated representation of the organizational communication network. A line from one node to another indicates that one or more emails were sent along that directed tie. Nodes are color-coded by their sub-organization

Our analyses capture communication as a network reflecting interactions among members of this organization. This network is represented as a series of *nodes* representing individuals and *ties* representing interactions between them. As this is an email communication network, it is inherently directional because the sender and receiver roles are distinct. One must actively initiate a communication act that forms the basis of a tie, but individuals passively receive ties. Distinguishing between the two types of ties is important, as it takes more effort to initiate a tie in this email communication network than to receive one. To reflect the differences between these two types of ties, we refer to *out-ties* as ties initiated by an individual and *in-ties* as ties received by the individual. Depending on which statistical model was used to characterize the network (more details on network models follow later in this section), ties can either represent discrete communication actions or interactions aggregated over time. The former representation captures a sequence of interactions over time while the latter captures a snapshot of the network over a given time period. Figure 1 shows a time-aggregated representation of the network. A tie from node *i* to node *j* indicates that *i* sent one or more emails to *j* during that timespan. This representation is useful for characterizing network structure over broader timespans, although aggregation necessarily reduces the resolution of the data. For example, the aggregated network cannot distinguish between a tie along which fifteen emails were sent and a tie along which only two were sent. Capturing the volume of traffic across ties is feasible by representing the network as a time series of discrete interactions, where each interaction from *i* to *j* is represented as a distinct, directed tie. This approach captures information encoded in the timing and sequencing of interactions. This paper models both representations to capture both structural and temporal features of the network.

### Measuring trust

During the training exercise, participants regularly completed surveys about their knowledge and experiences during the scenario. We focus specifically on their regular reports of trust in various groups within the organization. For each of the three organizations—the US Brigade, the UK. Brigade, and the Division—individuals responded to the question “How likely is it that individuals from the following groups will contribute to mission success?” Individuals rated all three, including their own group, on a scale from 0, indicating that the group was not at all likely to contribute to the organization’s success, to 10, indicating certainty that the group would contribute to organizational success. This measure of trust adheres to the Robinson (1996, p. 576) definition of trust as a measure of “expectations, assumptions, or beliefs about the likelihood that another’s future actions will be beneficial, favorable, or at least not detrimental to one’s interests.” This measure captures the cognitive and behavioral foundations of trust discussed by Lewis and Weigert (1985), where one’s assessment of trust in another individual or group relies on some knowledge about the referent’s propensity to take a behavioral action (here, contributing to organizational success). These assessments may reflect experiences interacting with the group in the past or may portend future interaction, suggesting the usefulness of a time-resolved, network-based approach to understand trust within an organization.

Participants rated organizational trust at multiple time points, enabling the opportunity to examine trust dynamics. The training exercise took place over the span of approximately ten days in the operational environment. Due to the simulated operational environment, however, this ten-day span was compressed such that each “day” within the training scenario spanned approximately 6 h in real time. These ten days were split into two weeks, such that participants completed the first week (five simulated days in the training environment) and had several days off before resuming the second week of the exercise. Participants completed the trust survey at the end of each of these six-hour days, except for the final day in each week. Accordingly, each individual provided trust ratings for each group eight times over the course of the training exercise. These repeated measures capture how trust within and across groups waxes and wanes over relatively short time scales during this training exercise.

Surveys showed that the UK group received the highest trust ratings followed by the US and the Division. These rankings were consistent with the rankings of ratings provided by members of each group, where the UK gives the highest trust ratings followed by the US and the Division. Table 1 shows each group’s trust ratings averaged across all respondents and all eight time points. On average, the Division reports the lowest level of trust in itself, in contrast to the US and UK groups that each give the highest trust ratings to themselves.

**Table 1:** Average inter- and intra-group trust ratings

	Div.	US	UK
Div	7.79	8.18	8.34
US	7.86	8.44	8.05
UK	8.07	7.91	8.67

Trust ratings varied over time and showed no pattern of convergence. Averaged across all respondents in all three groups, trust ratings peaked at 8.06 in the first survey before reaching a nadir of 7.72 on the fifth survey and then steadily recovering to 7.79 by the eighth and final survey. This rebound does not approach the trust ratings reported during the first simulated week in the training exercise, as four of the five highest average ratings for trust occur during the first week. Volatility of trust ratings grows over the course of the exercise, as the standard deviations for all trust ratings begin at 1.94 for the first survey, climb to 2.02 on the second survey, hover between 2.11 and 2.16 during the third through sixth surveys, and climb to 2.25 and 2.40 on the last two surveys, respectively.

**Fig. 2:**
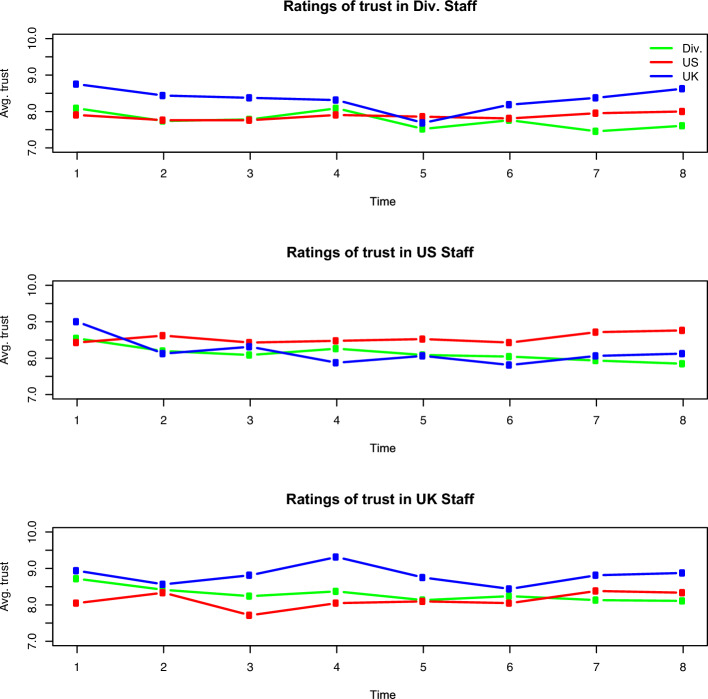
Trustworthiness ratings for each organization over time

Figure 2 illustrates the average of each group’s rating for each other group over the course of the two-week exercise. Each of the three plots features a group as the referent of trust and the three color-coded lines indicate each group’s average rating of trust in that referent group. The UK consistently reports the highest trust in itself and the Division and it reports the lowest trust in the US group.

## Modeling intra-organizational communication

We use three types of network models to characterize the relationship between trust and communication, net of several control terms. The first approach models the structural configuration of the network to determine if an individual’s trust in an organization predicts communication ties to members of that organization. The second modeling framework models evolution of the network across multiple, time-aggregated snapshots to assess if trust in an organization predicts either formation, persistence, or dissolution of ties to members of that organization. Finally, the third modeling framework captures the rates of interactions to capture whether trust in an organization predicts the *rate* of interactions to members of that organization. Measures of trust serve as the crux for these analyses and we briefly discuss our approach for measuring individual trust in each group. From each individual we have three measures of trust at each of the eight time periods: trust in the UK Brigade, trust in the US Brigade, and trust in the Division. From these we construct a matrix with a row and column for every individual in the organization where the *i*, *j* cell indicates individual *i*’s trust in individual *j*. Because asking each of the organization’s 83 members to report his or her trust in every single other individual would be an excessive burden, surveys instead asked about trust in *groups* within the organization. Thus for each *i*, *j* cell *i*’s reported trust in *j* represents *i*’s trust in *j*’s *group*. If *j* belongs to the UK Brigade and *i* gives the UK Brigade a trust rating of 7, then we treat *j* and all other members of the UK Brigade as if *i* gave them ratings of 7. From each of the 83 members, therefore, we have ratings of their trust in others based on group membership; and we have these ratings for 8 time periods. We refer to a matrix of trust ratings as $\tau$. To align communication with each of these trust ratings, we bin communication logs such that they cover time between trust surveys. The first bin covers the start of the exercise until the first trust survey, the second covers all communication after the first trust survey until the second trust survey, and so on. Each of those eight time periods provides an opportunity to examine the relationship between the communication network and trust ratings.

Trust may be a product of or a precursor to communication (or it may be entirely unrelated). Trust reported at one time point may shape communication at the following time point, or that trust may reflect patterns of communication preceding those trust ratings, per the intuition for Hypotheses 1a and 1b. These hypotheses, respectively, capture whether trust is a product of communication or an antecedent to communication. Additionally, the change in trust over a particular time period may shape communication during that time period (or vice versa). To capture these different relationships, we use three measures of trust. $\tau _t$ refers to trust measured at the end of time *t*; we will use this measure to determine if trust reported at the end of time *t* is a product of observed communication *during* time *t* (Hypothesis 1b). $\tau _{t-1}$ refers to trust measured at the end of the *previous* period of communication; this will demonstrate whether trust at time $t-1$ forecasts communication at time *t*. These two measures will allow us to disentangle whether communication forecasts future trust or whether trust forecasts future communication. Finally, $\tau _{\Delta }$ refers to the *change* in trust from time $t-1$ to time *t*. Although this does not pertain to one of the hypotheses, this measure will determine if a change in trust from $t-1$ to *t* has any relation to the communication network during time *t*. The remainder of this paper examines how these different measures of trust predict network structure and flows across ties in the communication networks.

### ERGM

The first of the three families of network models captures the presence of ties in the observed network. The exponential-family random graph model (ERGM) estimates the effects of current trust $\tau _t$, prior trust $\tau _{t-1}$, or a change in trust $\tau _{\Delta }$, net of several control terms, on the static communications networks. In these analyses, each communication network represents a time-aggregated representation of the email logs, where an *i*, *j* tie exists if *i* sends one or more emails to *j* during the observed period of time. ERGMs use properties of an observed network to parameterize a probabilistic model (Robins et al., 2007). Equation 1 illustrates the exponential-family form below.

1 $$\begin{aligned} P(Y=y) = \frac{exp(\theta 'g(y))}{\kappa (\theta )} \end{aligned}$$

In Equation 1, *Y* represents a random variable for the state of the network with realization *y*, the observed network. *g*(*y*) represents a vector of sufficient statistics (such as node attributes, tie attributes, or features of network structure represented in the model’s control terms) for the network and $\theta$ represents a vector of coefficients for those sufficient statistics. Their product is divided by $\kappa (\theta )$, a normalizing factor that represents the numerator summed over all possible configurations of the observed network. The model tunes the coefficients to maximize the likelihood of the observed network to demonstrate whether each of the sufficient statistics is more or less likely than expected by chance (where chance represents all the other configurations this network could have taken).

The principal challenge in computing ERGMs is calculating all possible network configurations needed by $\kappa (\theta )$, and this poses unique challenges for addressing traditional statistical issues such as statistical power. Network structure is driven by exogenous factors—node attributes such as unit or rank—and endogenous factors—dyadic dependence, or autocorrelation in the network’s underlying adjacency matrix where, for example, a reciprocal tie between nodes *i* and *j* cannot exist unless *i* directs a tie to *j* *and* *j* directs a tie to *i*. Due to dyadic dependence, ERGM treats the entire network as a single observation rather than as a series of independently observed ties, as some model terms (e.g., reciprocity) render sets of ties inextricably dependent upon one another. As ERGM treats the network as a single observation, it cannot derive statistical power from the size of the sample of individuals represented in the network, as is common in regression analyses. Rather, in the ergm package (Hunter et al., 2008; Handcock et al., 2018) in the R statistical computing environment, ERGM uses simulation to build a sufficient sample to ensure adequate statistical power (Cao et al., 2024; Kolaczyk & Krivitsky, 2015). The ERGM algorithm’s built-in functionality uses Markov Chain Monte Carlo (MCMC) sampling to simulate the space of possible networks given the number of nodes in the observed network. MCMC simulation is necessary because generating all possible networks to calculate the exact likelihood function is computationally intractable for all but the smallest networks. Given that the networks in these analyses have 83 nodes, 6,806 unique ties are possible (assuming self-ties are not feasible), leading to approximately 8.57 *x* $10^{1932}$ possible network configurations. Simulating a sample of possible network configurations enables the estimation of the approximate distribution of a given network statistic without enumerating all possible networks (Cranmer & Desmarais, 2011), thus facilitating the maximum likelihood calculation. ERGMs were calculated using the ERGM function’s default sample size of 4,096 and STERGM models used the default sample size of 1,024. The model’s convergence on a set of coefficient estimates demonstrated that the models avoided issues of model degeneracy and obtained reliable sets of coefficients explaining the observed networks, and this lack of degeneracy was confirmed by examining the distribution of model statistics over time during the MCMC procedure.

### STERGM

Moving beyond a static representation of this email communication network, the next set of models examines evolution of the network from one time point to the next. An extension of ERGM, the separable, temporal ERGM (STERGM) models dynamic networks in discrete time across multiple time points (Krivitsky & Handcock, 2014). STERGM differs from ERGM in its separable parameterization, which produces a pair of joint models: One models tie formation over time and the other models tie persistence over time. Modeled together within STERGM, formation and persistence occur independently during the same period of time according to the model’s assumptions. These models were fit with the tergm package (Krivitsky & Handcock, 2016) in R. Equation 2 demonstrates the formation model and its near-identical counterpart in Equation 3 demonstrates persistence.

2 $$\begin{aligned} ln \frac{P(Y_{ij,t+1}=1|y^c_{ij}, Y_{ij,t}=0)}{P(Y_{ij,t+1}=0|y^c_{ij}, Y_{ij,t}=0)} = \theta ^+\delta (g^+(y))_{ij} \end{aligned}$$

3 $$\begin{aligned} ln \frac{P(Y_{ij,t+1}=1|y^c_{ij}, Y_{ij,t}=1)}{P(Y_{ij,t+1}=0|y^c_{ij}, Y_{ij,t}=1)} = \theta ^-\delta (g^-(y))_{ij} \end{aligned}$$

Both STERGM frameworks operate similarly although they necessarily focus on distinct aspects of network dynamics. The formation model accounts for tie emergence by positing the log-odds of an *i*, *j* tie at time $t+1$ given all other ties ($y^c_{ij}$) at time *t* *and* given that an *i*, *j* tie does not exist at time *t*. Positing the likelihood of a tie emerging where one did not exist in the preceding time point, this necessarily models tie formation. $g^+(y)_{ij}$ represents a vector of sufficient statistics in the formation network and $\theta ^+$ represents a vector of coefficients for those sufficient statistics. Using maximum likelihood estimation to find the coefficients that maximize the probability of the realized formation network, this model closely mirrors the ERGM framework described above. The persistence model operates identically to the formation model, but it posits the likelihood of an *i*, *j* tie at time $t+1$ given that an *i*, *j* tie *already exists* at time *t*. It therefore captures processes of tie persistence.

This model framework allows us to examine whether our observed trust measurements shape network evolution over time, net of our control terms. The effect for current trust ($\tau _t$) indicates whether trust at time *t* predicts formation of a tie between times $t-1$ and *t* or persistence of a tie across those time points; a higher coefficient for this term indicates that higher trust in a group at time *t*, respectively, predicts increased likelihood of formation or persistence of ties to members of that group. Prior trust ($\tau _{t-1}$) flips the temporal direction of this relationship by capturing whether trust at $t-1$ then predicts the likelihood of tie formation or persistence between $t-1$ and *t*; a higher coefficient will indicate that higher ratings of trust during the previous time period predict increased likelihood of subsequent tie formation or tie persistence, respectively, during the current time period. The distinction between $\tau _t$ and $\tau _{t-1}$ lies in the temporal order: Does prior trust forecast subsequent communication or does communication forecast the subsequent rating of trust? Finally, an edge term for $\tau _{\Delta }$ indicates whether a change in trust between $t-1$ and *t* is associated with tie formation or persistence. A positive coefficient for this final term indicates and an increase in trust in a group will be associated with increased likelihood or forming or sustaining ties to members of that group.

### Relational event models

The previous two modeling approaches treated each snapshot of the network as a time-aggregated representation of communication. The next modeling framework, the relational event model, preserves the original format of the communication data by accommodating a series of time-ordered, discrete communication acts. The relational event model posits that the likelihood of a relational event *a*, in which *i* interacts with recipient *j* at time *t*, arises as a function of the history of past events and attributes for both events and individuals (Butts, 2008). This relational event *a* is part of a larger event history $A_t$, which we ultimately aim to model. The conditional likelihood for the *i*th event in our history $A_t$ at time $t(a_i)$ is equal to the hazard for relational event $a_i$. This hazard represents the likelihood that this event transpires at time $t(a_i)$ given that it has not already transpired, multiplied by the survival functions for all potential relational events over the time interval from the previous event to the current event. That is, this represents the likelihood that this particular event transpires rather than some other event transpiring or no event at all transpiring since the last event. The joint likelihood for the *entire* event history $A_t$ is a product of each event’s conditional likelihood and the likelihood of no events occurring between the final event and the end of observation time. As this is a piecewise constant latent hazard model, each potential event has a constant hazard of occurrence given the prior event history (this hazard is updated as each new event becomes incorporated into the prior event history). More simply, the probability of a particular event is its occurrence rate divided by the sum of rates of all possible events.

The relational event model richly captures the effects of time on network dynamics. Many communicative actions depend on temporal context (e.g., turn taking, recency bias) that is lost when aggregating network data over time. Capturing the richness embedded in this temporal context will transform our understanding of how group states and processes evolve. Emphasizing the immense potential of the relational event model on group research, Leenders et al. (2016, p. 97) states that the “holy grail for research on team dynamics is to be able to watch a ‘movie’ of team process as it unfolds, then pause the movie, and be able to answer the questions: What will likely happen next and with what implications for the outcomes?” By answering what will happen next, the relational event model gives us a foundation to be able to achieve this “holy grail” described by Leenders and colleagues. Indeed, the relational event model has already been used to improve our understanding of group behavior (Pilny et al., 2014; Quintane et al., 2014; Schecter & Contractor, 2017; Schecter & Quintane, 2021).

Complementing the ERGM and STERGM approaches, which reveal how current trust, prior trust, or change in trust shape the presence or absence of ties, the relational event modeling approach evaluates the *rate* of communication across a tie as a function of the trust rating on that tie. We use an edge covariate effect to capture the propensity for an individual’s trust in a group shapes the rate of interaction to members of that group. The coefficient for current trust ($\tau _t$) indicates the relationship between trust in a group at time *t* and the rate of communication to members of that group during time *t*. The effect for prior trust ($\tau _{t-1}$) indicates whether trust reported at $t-1$ shapes the rate of communication during time *t*. Lastly, the effect for $\tau _{\Delta }$ demonstrates the relationship between change in trust in a group from $t-1$ to *t* and the rate of communication to members of that group during *t*. In a relational event context these edge effects for trust illustrate the relationship between trust and communication rates.

## Results

Table 2 shows the volumes of ties analyzed in the ERGMs and numbers of emails analyzed in the relational event models (the STERGM subsection lists the formation and persistence statistics for the STERGM analyses). Although network size necessarily remains fixed due to stable organizational membership, the volume and structure of communication vary across each time period. The number of emails varies nearly by an order of magnitude between the least active (Time 1) and most active (Time 5) periods. Varying 3.5-fold between the most and least active time periods, the total volume of ties shows less variation than the number of emails. Density represents the proportion of ties realized in the network, while mean degree represents the average sum of in-ties and out-ties in the network. Both measures are directly proportional to the number of ties. The average number of emails sent along each tie ranges from fewer than two per tie (Time 1) to over four per tie (Times 3 and 5). A strong, positive correlation emerges between the volume of emails per tie and the mean degree (r=.85), indicating that larger volumes of communication coincide with greater breadth of communication channels, rather than increased utilization of the same communication channels.

**Table 2:** Descriptive statistics for each of the eight networks

	Emails	Nodes	Ties	Density	Mean degree
Time 1	419	83	231	0.034	5.566
Time 2	1,021	83	404	0.059	9.735
Time 3	2,722	83	639	0.094	15.398
Time 4	1,405	83	384	0.056	9.253
Time 5	3,292	83	807	0.119	19.446
Time 6	830	83	352	0.052	8.482
Time 7	726	83	327	0.048	7.880
Time 8	1,327	83	484	0.071	11.663

Before proceeding through the battery of trust results, we begin with an illustrative model demonstrating the set of terms included in the ERGM and STERGM analyses. All sets of ERGMs and STERGMs were fit with the same model terms to facilitate comparison of trust results across time period and across trust type (prior trust, current trust, and trust change). The goodness-of-fit approach of Hunter et al. (2008) validated the adequacy of these models’ fit to the observed data. This approach uses each model’s coefficients to simulate 100 networks from the model and compares those simulated networks’ structure to that of the observed network to ensure that the model produces an adequate representation of the underlying data. For ERGM, this entails reproduction of the original network, while STERGM’s joint models produce tie formation networks and tie persistence networks. All the models in our results provided accurate reproductions of their respective network statistics (including in- and outdegree distributions, edgewise shared partner distributions, and geodesic distances) and thus accurately reproduce the structure of the observed networks. The following illustrative ERGM assesses network structure during the time preceding the second trust survey.

**Table 3:** ERGM model coefficients (Time 2)

Term	Coefficient	Standard error	Odds ratio
Baseline tie occurrence	$-$5.59***	0.40	0.00
Prior trust ($\tau _{t-1}$)	0.10*	0.05	1.10
Trust propensity in-ties	0.07*	0.03	1.07
Trust propensity out-ties	$-$0.09*	0.05	0.91
UK	0.04	0.08	1.03
US	$-$0.01	0.09	1.00
Same-cell ties	0.66***	0.13	1.91
Coordinative role in-ties	0.26	0.15	1.28
Coordinative role out-ties	$-$0.12	0.15	0.88
Rank in-ties	$-$0.05	0.03	0.96
Rank out-ties	0.10**	0.03	1.10
SA in-ties	$-$0.51	0.53	0.63
SA out-ties	0.35	0.55	1.44
Tie inertia	1.26***	0.10	3.51
Reciprocity	1.26***	0.22	3.51
Transitivity	1.19***	0.08	3.30
Null deviance	9345	(df=6806)	
Residual deviance	1978	(df=6789)	

*p < .05       **p < .01       ***p < .001

The model contains three trust-related terms and numerous control terms to account for other drivers of network structure. The first trust term in Table 3 captures prior trust ($\tau _{t-1}$), the measure of inter-organizational trust conducted at the previous time period. This model shows a positive, significant effect, indicating that trust ratings from the prior time period positively predict communication ties during the present time period. If an individual reported a one-unit increase in trust in the UK group compared to other groups during the previous time period, he or she will be 1.10 times more likely to send a tie to someone in that group, net of the other model terms. The "trust propensity" node attribute effects account for individual variation in propensities to trust others (Gurtman, 1992; Sorrentino et al., 1995; Kramer, 1999), and control for how individual propensities to trust may drive their tendencies to send or receive ties. Trust propensity was calculated as the average of each individual’s trust ratings for all groups over the entire exercise. This model shows that those who provide higher ratings of trust tend to have more in-ties and fewer out-ties than expected by chance, indicating lower rates of initiating communication ties and greater rates of attracting communication ties. Compared to a typical individual, an individual reporting a one-unit higher rating in average trust ratings will receive 1.07 times more in-ties but send only 0.91 times as many out-ties. Beyond this illustrative model, about half the ERGMs find negative, significant out-tie effects for trust propensity, and about one-quarter of models find positive in-tie effects for trust. These results indicate that individuals who tend to give higher-trust ratings frequently send fewer ties than expected by chance and occasionally receive more ties than expected by chance.

### Model controls

In addition to the trust terms, the model controls for potential confounding variables by including a large number of individual attributes and features of network structure that may drive the underlying structure of the communication network. Controlling for these helps to isolate trust-driven effects from other factors such as familiarity, organizational roles, or situation awareness. In many cases, individual attributes driving the initiation of communication are distinct from attributes driving the attractiveness of an individual as a communication partner. As such, control terms for many individual attributes are separated into their out-tie, or sending, effects and their in-tie, or receiving, effects. The next several paragraphs walk through each of the model terms and provide theoretical justification for their incorporation into the model.

**Organizational Membership:** The first set of control terms accounts for elements of the organization itself that may drive network structure. The “US” and “UK” terms, respectively, capture whether US or UK group members have more or fewer ties than expected by chance, relative to members of the Division. In this example no effect emerges for either group. This finding was typical of the first week of the exercise, while they wavered between positively and negatively significant during the second week.

**Subgroup Membership:** Individuals performing similar functions in an organization tend to be more likely to communicate with each other. The “same-cell ties” term captures the tendency for members within a functional cell (e.g., intel, sustainment) to form ties to fellow members of that cell; the positive effect here indicates an elevated propensity for individuals to communicate with other members of their functional cell. This strong, positive effect is consistent across all models for all time periods, as many communication partners have a shared organizational function.

**Coordinative Roles:** Many organizations establish roles that function to coordinate and facilitate information exchange across the organization (Galbraith, 1977; Marks et al., 2001; Van de Ven et al., 1976). These roles may play an important role in shaping trust within the organization, as such roles “function to reduce uncertainty regarding role occupant’s trust-related intentions and capabilities” (Kramer, 1999, p. 578). Fulfillment of one of these roles may lead an individual to have a higher propensity to be seen as a trustworthy communication partner, and controlling for such roles is thus necessary. The “coordinative roles” terms capture the tendency for individuals in coordinative roles to send or receive ties. In addition to occupying a role in a functional cell, eight members of the organization occupy roles in the “integrating cell” (2 in the UK group, 1 in the US, and 5 in the Division). Individuals in this cell collect and disseminate information about current operations and upcoming orders and plans. This model shows no significant in-tie or out-tie effect for coordinative roles. Models from other time periods tend to find negative in-tie effects and positive out-tie effects, indicating a tendency for coordinative roles to initiate more communication ties and receive fewer communication ties than expected by chance.

**Rank:** In organizational contexts, individuals tend to seek out others with rare or valuable knowledge (Carley & Hill, 2001), and this model uses two sets of terms to account for potential indicators of that attractiveness to potential communication partners, rank, and situation awareness. The above illustrative results show that those with higher rank tend to be more likely to send ties, but they are no more likely than chance to be the recipients of ties. Models from other time periods consistently find positive out-tie effects for rank and significant, negative in-tie effects for rank in about half of the models. Higher-rank individuals typically initiate more ties and occasionally receive fewer ties than expected by chance. Further investigation of the rank term found that terms accounting for rank *differences* showed that ties more commonly went from higher-ranking individuals to lower-ranking individuals. However, models with the rank difference term did not achieve better fit than models with the in-tie and out-tie effects and were thus not included in the full results.

**Situation Awareness:** The next two control terms account for individuals’ situation awareness (SA), as SA may shape an individual’s propensity to send or receive ties. SA was measured with the Situation Awareness and Global Assessment Technique (SAGAT) (Endsley, 1988, 1995, 2000). This technique measures individual situation awareness (SA) by periodically asking respondents about features of their task environment, not unlike a pop quiz. This has been implemented in a wide range of organizational contexts, including medical care (Wright et al., 2004), team training (Patrick et al., 2006), and military operations (Salmon et al., 2006). Once during each simulated day individuals were asked questions such as “Do you currently have troops in contact?” and “In your sector, which of the following civilian activities are currently occurring?” We developed a measure of each individual’s SA over the exercise by aggregating their scores across all SA measures, as SA tends to be more reliably measured through assessment over several important tasks (Salas et al., 1995). This example model shows that an individual’s SA does not shape the propensity to send or receive ties. Other models show several negative out-tie effects for SA coupled with positive in-tie effects for SA. This reflects a tendency for high-SA individuals to be attractive targets for communication, although they tend to avoid initiating communication, perhaps because the act of initiating many communication ties may be at odds with their ability to maintain a strong understanding of their task environment (Fitzhugh et al., 2020).

**Tie inertia:** The next effect moves beyond nodal covariates to capture a covariate of the tie itself. The “tie inertia” term controls for the tendency for heavily utilized ties in the past to be more likely to be utilized in the future. Counting the number of emails sent along each tie prior to the observed period of communication, this term is positive when prior volumes of ties predict increased likelihood of observing that tie. For this model and nearly every ERGM, results show a positive, significant effect where greater utilization of a tie in the past predicts increased likelihood of utilization at present. That is, individuals are more likely to utilize stronger, pre-existing relationships than forge new communication channels.

**Reciprocity:** Reciprocity captures the tendency for individuals to reciprocate communication ties, such as *i* sending a tie to *j* and *j* reciprocating by sending a tie to *i*. This model finds a positive, significant effect for reciprocity; this effect holds in every time period except the fourth.

**Transitivity:** Nodes may be more likely to interact with their communication partners’ communication partners (e.g., a “friend of a friend is my friend“ effect). The final control term represents triadic closure, or the tendency for *i* to form a tie to *j* if *i* forms a tie to *k* and *k* forms a tie to *j*. This captures the tendency for ties to form among individuals with mutual communication partners. In this model, and all other ERGMs, results show a positive, significant effect for transitivity.

### ERGM trust results

**Fig. 3:**
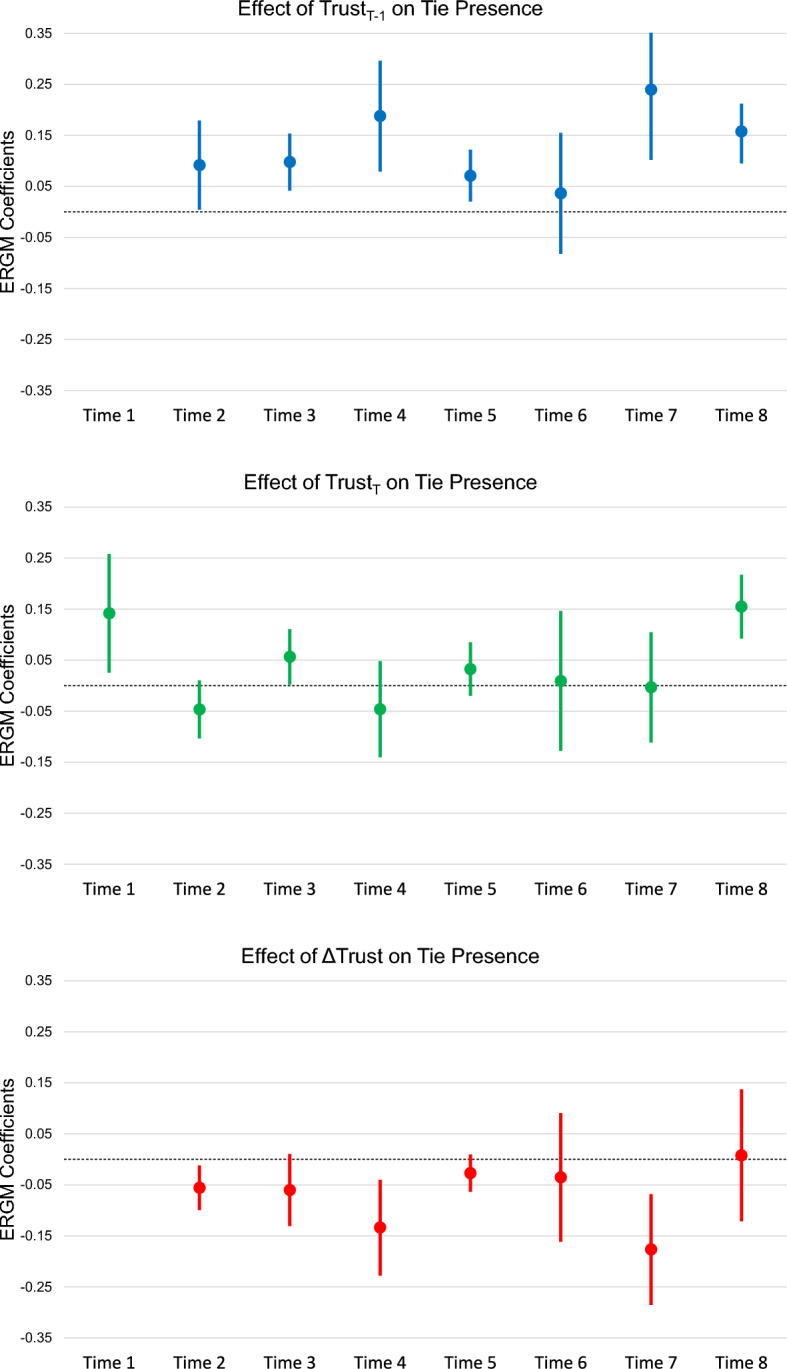
ERGM trust coefficients

To maintain continuity across time periods, ERGMs and STERGMs have identical model terms to the previous example. This helps to isolate trust effects from other factors potentially driving communication structure, such as organization role, rank, SA, or structural features such as reciprocity, inertia, or transitivity. Due to correlations between current trust ($\tau _t$) and prior trust ($\tau _{t-1}$) ranging from .65 to .94, and perfect collinearity if the model includes both those terms and trust change ( $\tau _{\Delta }$), separate models capture each trust effect of interest. This produces a total of 22 models (7 each for prior trust ($\tau _{t-1}$) and trust change ($\tau _{\Delta }$) and 8 for current trust ($\tau _t$)) for each of our three families of models. Prior trust and trust change have one fewer model because trust was not measured prior to the start of the exercise, thus there was no measure of prior trust or change in trust at the end of the first time period. To avoid overwhelming the reader with tables from 22 models, the full results tables reside in Appendix. The remainder of this section presents a more streamlined representation of the trust-based results beginning with the ERGM results. Figure 3 and Table 4 illustrate the ERGM coefficients and their corresponding standard errors for the three measures of trust.

**Table 4:** ERGM inter-organizational trust coefficients (see Appendix for full model tables)

	Prior Trust ($\tau _{t-1}$)		Current Trust ($\tau _t$)		Trust Change ($\tau _{\Delta }$)	
	Coef.	SE	Coef.	SE	Coef.	SE
Time 1			0.153**	0.059		
Time 2	0.092*	0.046	$-$0.048	0.030	$-$0.056*	0.022
Time 3	0.095**	0.029	0.046	0.029	$-$0.063	0.033
Time 4	0.0191***	0.057	$-$0.052	0.045	$-$0.132**	0.045
Time 5	0.116**	0.040	0.045	0.037	$-$0.020	0.030
Time 6	0.038	0.060	0.014	0.073	0.013	0.074
Time 7	0.242**	0.074	$-$0.007	0.057	$-$0.173**	0.061
Time 8	0.246***	0.057	0.272***	0.053	0.220***	0.082

***p <.001,      **p < .01,     *p < .05

Utilizing multiple measures of trust provides unique insight into the temporal directionality of the relationship between trust and communication. Six of seven ERGMs show positive, significant effects for prior trust ($\tau _{t-1}$) on communication. This demonstrates that when individuals report higher trust in a group, they are more likely to decide to communicate with members of that group during the next time point. By contrast, only two of the eight ERGMs show a positive, significant effect for current trust ($\tau _t$). This provides limited evidence that communication with members of a group during a given time interval predicts increased reported levels of trust in that group at the end of that time interval. Together these findings suggest that trust much more often serves as a forecast of communication channels rather than an outcome. These results provide strong evidence for Hypothesis 1a (trust predicts a subsequent increase in communication) and limited evidence for Hypothesis 1b (communication predicts a subsequent increase in trust). Providing some further evidence that trust does not necessarily result from communication ties to a group, trust change ($\tau _{\Delta }$) produces a negative effect in three of the seven ERGMs. A negative, significant $\tau _{\Delta }$ indicates that when individuals *increase* their ratings of trust in a group during the current time period, they are *less likely* to decide to form ties to that group. Likewise, individuals who lower their reported ratings of trust in a group tend to have more ties to that group. This may reflect that as trust in a group declines, individuals decide to cast a broader net to find reliable communication channels to that group. By probing different time-dependent measures of trust these findings provide some evidence that communication does not always function as a precursor to trust; rather, trust consistently serves as a prerequisite to communication.

### STERGM results

The next set of model results examines evolution of the communication network from one time point to the next. While the ERGM framework posits the effect of various network statistics on the presence or absence of a tie, the STERGM framework accommodates four potential tie states: A dyad (that is, any given pair of nodes) with no tie remains without a tie, a dyad without a tie forms a tie, a dyad with a tie sustains that tie, and a dyad with a tie dissolves that tie. By incorporating information on the previous state of dyads, the STERGM framework evaluates the effect of trust on the evolution of the communication network. Table 5 presents the counts of ties formed, sustained, and dissolved across each of the time periods. Each of these dynamic processes depends on the immediately preceding time period. Formation of a tie from *i* to *j* requires that no *i*, *j* tie existed at the previous time period, even if *i* formed a tie to *j* several time periods prior. For example, if *i* forms a tie to *j* during $\hbox {T}_2$, dissolves that tie during $\hbox {T}_3$, and then creates another tie to *j* during $\hbox {T}_4$, we consider that to be tie formation from $\hbox {T}_3$ to $\hbox {T}_4$ as no tie existed during $\hbox {T}_3$. As such, the same tie may be formed, sustained, and dissolved multiple times.

**Table 5:** Communication tie dynamics

Time period	Ties formed	Ties sustained	Ties dissolved
$\hbox {T}_1$ to $\hbox {T}_2$	273	131	100
$\hbox {T}_2$ to $\hbox {T}_3$	366	273	131
$\hbox {T}_3$ to $\hbox {T}_4$	151	233	406
$\hbox {T}_4$ to $\hbox {T}_5$	530	277	107
$\hbox {T}_5$ to $\hbox {T}_6$	82	270	537
$\hbox {T}_6$ to $\hbox {T}_7$	157	170	182
$\hbox {T}_7$ to $\hbox {T}_8$	277	207	120

**Fig. 4:**
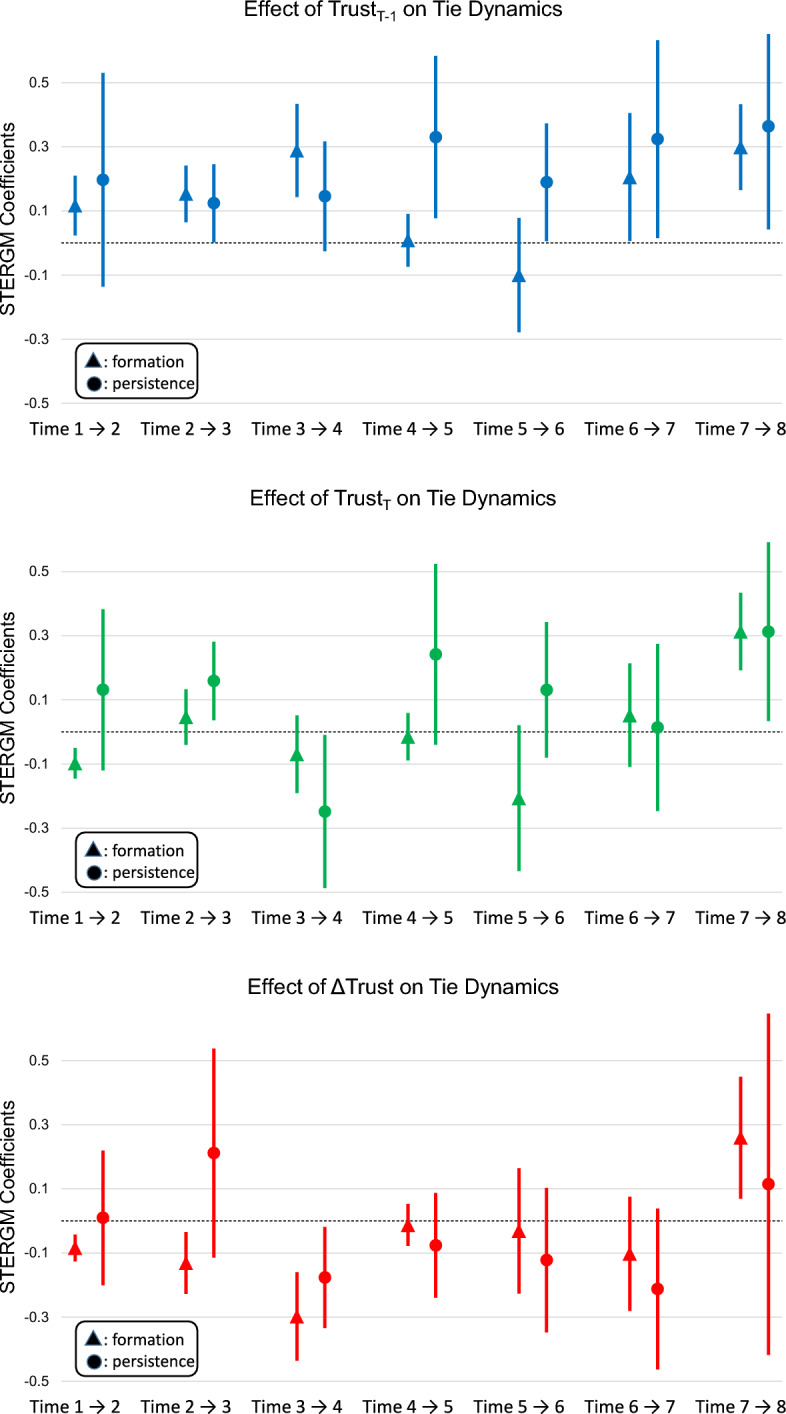
STERGM formation (left) and persistence (right) trust coefficients

Following the ERGM modeling strategy, these models have identical sets of terms. We again relegate the full results tables to Appendix to avoid excess clutter, and turn our attention to the trust-related coefficients. Figure 4 and Tables 6 and 7 show the results of inter-organizational trust on tie dynamics.

**Table 6:** STERGM formation inter-organizational trust coefficients (see Appendix for full model tables)

	Prior trust ($\tau _{t-1}$)		Current trust ($\tau _t$)		Trust change ($\tau _{\Delta }$)	
	Coef.	SE	Coef.	SE	Coef.	SE
Time 1–2	0.118*	0.050	$-$0.097***	0.029	$-$0.085***	0.021
Time 2–3	0.152***	0.045	0.046	0.042	$-$0.134**	0.044
Time 3–4	0.287***	0.076	$-$0.071	0.062	$-$0.299***	0.070
Time 4–5	0.008	0.042	$-$0.013	0.040	$-$0.015	0.033
Time 5–6	$-$0.105	0.094	$-$0.205	0.118	$-$0.032	0.105
Time 6–7	0.206*	0.103	0.056	0.085	$-$0.099	0.092
Time 7–8	0.298***	0.067	0.313***	0.061	0.261**	0.093

***p <.001,      **p < .01,     *p < .05

**Table 7:** STERGM persistence inter-organizational trust coefficients (see Appendix for full model tables)

	Prior trust ($\tau _{t-1}$)		Current trust ($\tau _t$)		Trust change ($\tau _{\Delta }$)	
	Coef.	SE	Coef.	SE	Coef.	SE
Time 1–2	0.195	0.167	0.132	0.128	0.012	0.109
Time 2–3	0.124*	0.062	0.159*	0.063	0.211	0.166
Time 3–4	0.145	0.085	$-$0.253*	0.122	$-$0.177	0.080
Time 4–5	0.330**	0.128	0.240	0.143	$-$0.076	0.083
Time 5–6	0.188*	0.094	0.138	0.105	$-$0.123	0.117
Time 6–7	0.326*	0.156	0.016	0.135	$-$0.213	0.123
Time 7–8	0.364	0.165	0.311*	0.140	0.118	0.272

***p <.001,      **p < .01,     *p < .05

Following the pattern established by the ERGM results, 5 of 7 models show that prior trust ($\tau _{t-1}$) produces a positive, significant effect for tie formation and 4 of 7 show a positive effect for tie persistence. When reporting higher levels of trust in an organization, individuals form new ties and sustain existing ties to members of that organization at higher rates than expected by chance. These results show that trust forecasts both creation and persistence of communication channels, thus providing support for Hypothesis 1 and building on the ERGM findings. Current trust ($\tau _t$) is associated with one positive effect and one negative effect for tie formation and two positive effects and one negative effect for tie dissolution. These results provide mixed and extremely limited evidence that tie dynamics during a given time span predict trust ratings at the end of that same time span. Finally, trust change ($\tau _{\Delta }$) has a negative effect on tie formation during the first three time periods and a positive effect during the final time period. The former results demonstrate that increased trust in an organization results in fewer ties formed to that organization in subsequent time periods; combined with the persistence findings, this may suggest that individuals become less likely to form higher-trust ties once they establish a quorum of reliable ties. A single negative effect for trust change ($\tau _{\Delta }$) is the only significant trust effect in the persistence models. The pattern of results is consistent with the ERGM results, with further evidence that increased trust ratings predict subsequent interactions rather than interactions predicting subsequent increases in organizational trust.

### Relational event model results

While the preceding two sections examined the *structure* of communication networks in static and dynamic realizations, this final results section uses relational event models to characterize the *rate of interactions* in these networks. This addresses the competing pair of hypotheses under Hypothesis 2 by determining whether elevated trust in a group is associated with enhancing or degrading the rate of communication to members of that group. Greater trust may predict enhanced rates of communication because trust facilitates information exchange and productive problem solving. Or trust may degrade communication rates because high-trust groups have enough understanding of others’ responsibilities that they can achieve coordinated task execution with minimal information exchange, in contrast to low-trust groups that communicate excessively to double-check each other’s intentions and painstakingly extract meaning from communication acts. Table 8 enumerates the control terms in the relational event models, all of which were fit using the relevent package in R (Butts, 2008). By treating each communication act as a discrete, time-ordered event, the relational event approach capitalizes on the information encoded in the timing and frequency of communication acts. This builds an understanding of what drives the *rate* at which individuals utilize ties, thus augmenting the knowledge gained from ERGM and STERGM models about the *structure* of these communication ties.

**Table 8:** REM model coefficients (Time 8)

	Coefficient	Standard error	Relative hazard
Baseline sending	$-$9.05***	0.33	
Prior trust ($\tau _{t-1}$)	0.19***	0.03	1.21
Trust propensity receiving	$-$0.08***	0.02	0.92
Trust propensity sending	$-$0.29***	0.04	0.75
Coordinative role receiving	0.29***	0.08	1.34
Coordinative role sending	0.15*	0.07	1.17
Rank receiving	0.14***	0.02	1.15
Rank sending	0.25***	0.02	1.29
SA receiving	$-$0.47	0.30	0.62
SA sending	$-$1.05***	0.31	0.35
Indegree receiving	6.48***	0.60	655.14
Outdegree sending	1.54***	0.39	1.54
Recency receiving	1.71***	0.10	1.71
Recency sending	3.38***	0.11	3.38
Transitivity	0.07***	0.01	0.07
Cyclicity	$-$0.02**	0.01	$-$0.02
AB-BA	0.62	0.35	0.62
AB-BY	$-$0.32	0.33	$-$0.32
AB-XY	$-$1.20***	0.14	$-$1.20
AB-AY	4.15***	0.14	4.15
Null deviance	23014	(df=1326)	
Residual deviance	13447	(df=1307)	

*p < .05       **p < .01       ***p < .001

This example highlights interaction dynamics during the final day of the exercise. Like the ERGM and STERGM results, this model uses three terms to evaluate trust-related effects on interaction rates. Directly relevant to Hypotheses 2a and 2b, this model shows that higher levels of trust in a group during the seventh time period were followed by greater rates of sending emails to members of that group during the eighth time period. Translating the relational event model coefficient to a relative hazard demonstrates that individuals will be 1.21 times more likely to interact with members of a group that has a one-unit higher rating of trust compared to other groups. The model also evaluated effects of trust propensity, and results showed that individuals with higher propensities to trust tend to send and receive fewer emails during this time period, a finding replicated in most time periods. An individual reporting a one-point higher average trust propensity will initiate interactions only 0.75 times as often and receive interactions only 0.92 times as often compared to others.

As with the ERGM and STERGM analyses, the relational event model includes a variety of terms to control for organizational, cognitive, and structural factors that may drive interaction rates in the network. Like the previous models, these control terms may be separated into sender and receiver effects; these, respectively, determine that rates at which individuals initiate or receive interactions. There is considerable overlap with many of the terms in the ERGM and STERGM models, but the sequencing embedded in the underlying relational event data enables controls not possible (or relevant) in time-aggregated representations. The following section walks through those terms and their patterns of results across all models.

### Model controls

**Coordinative Roles:** The “coordinative role” terms capture the tendency for coordinators to receive or send communication at rates beyond chance. Both sender and receiver effects are positive and significant, demonstrating that individuals in such roles send and receive emails at greater rates than expected by chance. About half of the relational event models for other time periods show positive sender and receiver effects for coordinative roles, suggesting that coordinators occasionally heavily utilize their ties and serve as attractive targets for emails.

**Rank:** Higher-ranking individuals send and receive emails at higher rates than expected by chance. Results from the rest of the relational event models consistently find positive sender and receiver effects for rank, indicating that higher rank individuals tend to send and receive more emails.

**Situation Awareness:** The receiving effect for situation awareness is not significant, although higher SA individuals tend to send fewer emails (likewise, lower SA individuals send more emails). Other relational event models frequently find negative sender effects for SA and more sporadic negative receiver effects for SA, suggesting that those with elevated SA tend to initiate and receive fewer emails. This pattern may reflect that the effort required to initiate and sustain outbound interactions (Granovetter, 1985; Mariotti & Delbridge, 2012; Uzzi, 1997) is at odds with the cognitive resources needed to stay abreast of one’s surroundings, particularly in rapidly evolving operational settings.

**Broadcasting:** The next two terms investigate whether the rates of sending or receiving emails are driven by preferential attachment (Barabási & Albert, 1999; Watts & Strogatz, 1998), or the propensity for a node that sends or receives many interactions to continue sending or receiving interactions at disproportionately high rates (e.g., a "rich get richer" effect). “Outdegree sending” represents a broadcasting effect, where sending many prior emails leads to increased rates of sending subsequent emails; the positive effect in this model provides evidence of that broadcasting effect. This positive, significant effect holds for the remainder of the relational event models, demonstrating a centralized network where elevated rates of emails flow from a handful of individuals taking on information broadcasting roles.

**Popularity:** “Indegree receiving” evaluates preferential attachment for incoming interactions by capturing the tendency for individuals who have received many emails to continue receiving many emails; the positive effect in this model shows an accumulating popularity effect where a disproportionately small set of individuals receives emails at elevated rates. This effect holds in all other time periods, demonstrating the emergence of certain individuals whose rates of receiving emails suggest that they serve as information sinks.

**Recency:** The salience of one’s interaction with another may shape the rate of subsequent interactions between the pair. The pair of “recency” effects captures the salience of prior interactions on subsequent interactions. A positive *receiving* effect demonstrates that *i* is more likely to email to *j* if *j* recently sent an email to *i*. A positive *sending* effect shows that *i* is more likely to send an email to *j* if *i* recently sent another email to *j*. Every model shows strong, positive effects for both recency terms, although the sending effects tend to be stronger than the receiving effects. This demonstrates that salience plays a consistent role in driving the rate of email communications.

**Transitivity:** The next pair of terms captures facets of triadic closure, or how often *i* and *j* interact if *i* interacts with a third party (*k*) who then interacts with *j*. The “transitivity” effect shows that *i* interacts directly with *j* at higher rates when many emails are sent from *i* to *k* and from *k* to *j*. However, the “cyclicity” term shows that *j* interacts with *i* at *lower* rates if *i* sends many emails to *k* and *k* sends many emails to *j*. The remainder of the models consistently find positive effects for transitivity and negative effects for cyclicity, demonstrating that interactions flow at higher rates when moving away from the source and at slower rates when cycling back toward the initiator of the interaction.

**Participation Shifts:** The final quartet of control terms captures participation shifts, common norms related to speaker and receiver order during conversation (Gibson, 2003). Gibson enumerates six common sequences of interactions during conversation between specific individuals. Characterized by a discrete communication act from a sender to a receiver followed by a subsequent communication act involving the initial sender, initial receiver, and/or a third party, these participation shifts closely follow the relational event framework. These relational event models employ four of these participation shifts that may closely follow the temporal logic of email conversations. Two fall under Gibson’s “turn receiving” category. The first, AB-BA (per Gibson’s nomenclature), captures the tendency for A to interact with B followed by B interacting A. This example and most other models show strong, positive effects for AB-BA reciprocation. The second turn-receiving event, AB-BY, features A interacting with B followed by B interacting with Y, which illustrates B’s (temporary) role as a broker between A and Y. There is no such effect in this model, although other models tend to have a negative effect for this term. The third participation shift term, AB-XY, falls under “turn usurping” in which an A-to-B interaction is next followed by an interaction from X to Y, both of whom are not parties in the prior communication. This term is negative here and in nearly all other models, suggesting a tendency against multiple, simultaneous conversations in these email networks. Finally, the models include the “turn continuing” participation shift AB-AY, where A interacts with B and then A interacts with another party, Y. This effect is strong and significant here and in all other models, suggesting a tendency for individuals to relay information to others in rapid succession. These four terms help to control for norm-driven short-term dynamics driving interactions in the network.

### Relational event trust results

**Fig. 5:**
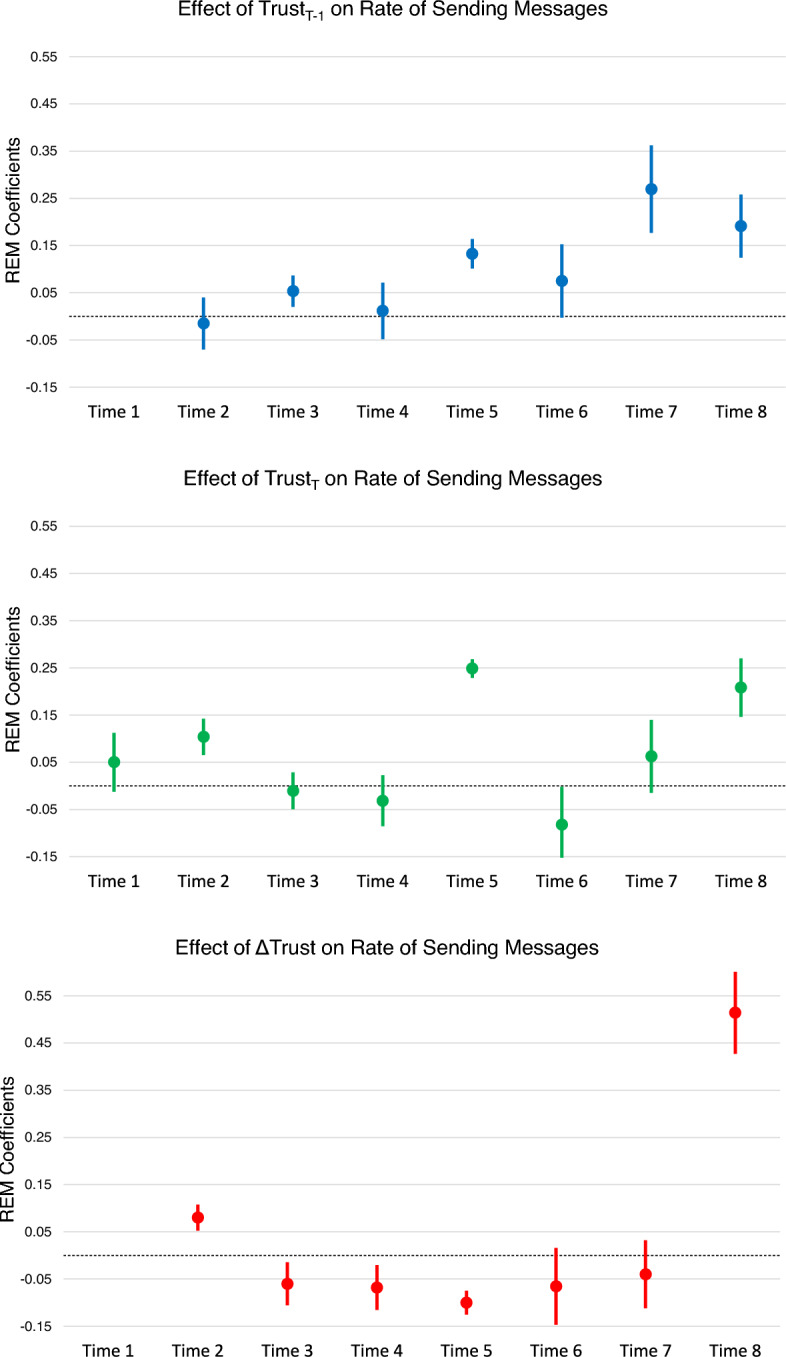
Relational event model trust coefficients

Relational event model trust results largely show consistency with the ERGM and STERGM results. Results consistently show negative sending and receiving effects for individuals’ trust propensity. That is, individuals who tend to give higher-trust ratings overall send and receive emails at rates lower than expected by chance. The tie covariates for trust assess the hypotheses pertaining to whether greater trust in a group is associated with increased or decreased rate of communication with members of that group. Figure 5 and Table 9 illustrate those coefficients for prior trust ($\tau _{t-1}$), current trust ($\tau _t$), and trust change ($\tau _{\Delta }$). Four of the seven relational event models find positive, significant effects for prior trust ($\tau _{t-1}$), indicating that trust in group members reported at the prior time point predicts increased rates of emails to those group members during the subsequent time period. Three models find positive, significant effects for current trust ($\tau _t$) and one model finds a negative effect. This provides some mixed evidence for the relationship between the rate of communication to members of a group during a given time period and trust in that group at the end of that time period. Trust change $\tau _{\Delta }$ also shows inconsistent results, as three negative, significant effects during the exercise are bookended by positive effects during the first and last time periods. These results indicate that positive changes in ratings of group trust correspond to increased rates of communication to those group members during some time periods and decreased rates during other time periods. Overall, the most consistent results provide moderate evidence that trust forecasts increased communication rates during the subsequent time period. This offers some support for Hypothesis 2a’s contention that increased trust is associated with increased interaction rates. Hypothesis 2b argued that increased trust would be associated with reduced rates of interaction; the lack of negative results for prior trust ($\tau _{t-1}$) (no significant effects) and current trust ($\tau _{t}$) (one significant effect) provide evidence to reject Hypothesis 2b. Beyond the association between trust and *rates* of communication, the discrepancy between $\tau _{t}$ and $\tau _{t-1}$ shows a temporal dependence in the relationship. Findings from the relational event models reflect the same general pattern observed in the ERGM and STERGM results in which higher trust in a group forecasts increased subsequent interactions with members of that group, while no such pattern emerges for increased interaction predicting subsequently higher trust ratings.

**Table 9:** Relational event model inter-organizational trust coefficients (see Appendix for full model tables)

	Prior trust ($\tau _{t-1}$)		Current trust ($\tau _t$)		Trust change ($\tau _{\Delta }$)	
	Coef.	SE	Coef.	SE	Coef.	SE
Time 1			0.050	0.032		
Time 2	$-$0.241***	0.020	0.104***	0.019	0.048***	0.013
Time 3	0.053**	0.017	$-$0.010	0.020	$-$0.060**	0.023
Time 4	0.012	0.030	$-$0.031	0.027	$-$0.083**	0.030
Time 5	0.133***	0.016	0.153***	0.009	$-$0.100***	0.013
Time 6	$-$0.069	0.041	$-$0.082*	0.041	$-$0.065	0.041
Time 7	0.269***	0.047	0.062	0.039	$-$0.039	0.037
Time 8	0.191***	0.034	0.208***	0.032	0.695***	0.028

***p <.001,      **p < .01,     *p < .05

### Results summary

Before proceeding to the discussion section, this subsection summarizes the extensive collection of results produced by the three distinct modeling approaches. Effects for prior trust ($\tau _{t-1}$) were more consistently positive than current trust ($\tau _t$) and trust change ($\tau _{\Delta }$), as they demonstrated that higher prior trust ratings predict increased communication in 5 out of 7 models for ERGM and STERGM-formation and 4 of 7 models for STERGM-dissolution and relational event models. Furthermore, each time point had at least one positive prior trust ($\tau _{t-1}$) effect across the models and all three models demonstrated unanimous effects for times 3, 5, 7, and 8. Results therefore find consistent but slightly curtailed support for Hypothesis 1a and Hypothesis 2a. When individuals report higher trust in a group, they subsequently engage in more communication ties to members of that group and higher rates of interaction across those ties.

When examining the effects of communication during the observed period on ratings trust at the end of that period (i.e., the current trust $\tau _t$ term), the results provide notably less consistent support. Positive effects for current trust ($\tau _t$) emerge in only 2 of 8 ERGMs, 1 of 7 formation STERGMs (along with a negative effect), 2 of 7 persistence STERGMs (along with another negative effect), and 3 of 8 relational event models (with yet another negative effect). These sparse findings provide limited and occasionally contrary evidence that increased interactions with members of a group during a given time period predict higher rates of trust in that group at the end of that time period. That is, while ratings of trust appear to forecast communication during the next time period, communication during a given time period does *not* consistently predict trust at the end of that time period. That is, there is limited evidence that communication to members of a group actively cultivates trust in that group.

Finally, effects of trust change ($\tau _{\Delta }$) also had limited impact on communication structure, dynamics, and rates. Negative effects for trust change ($\tau _{\Delta }$) emerged in 3 of 7 ERGMs (along with one positive effect), 3 of 7 formation STERGMs (along with one positive effect), 0 of 7 persistence ERGMs, and 3 of 7 relational event models (along with 2 positive effects). These suggest that an increase in trust in a group occasionally coincides with a simultaneous decrease in interactions with members of that group. The mixed and inconsistent results for current trust ($\tau _t$) and trust change ($\tau _{\Delta }$) show a stark contrast to prior trust ($\tau _{t-1}$), suggesting that trust more reliably forecasts subsequent communication structure and rates than such communication forecasts subsequent trust.

## Discussion

These findings contribute to theories of relational exchange by providing evidence for temporal directionality in the relationship between interactions and trust. This is particularly novel given the sparse understanding of trust dynamics (Serva et al., 2005; Lewicki et al., 2006; Zaheer & Harris, 2006). These results show that when individuals report trust in an organization, they become more likely to form communication channels to members of that organization and to utilize those channels at higher rates. This is consistent with findings that relationships characterized by enhanced trust facilitate increased information exchange and knowledge sharing during organizational problem-solving (Muthusamy & White, 2005; Zaheer & Harris, 2006; Murnighan & Conlon, 1991). However, these findings build on those prior theories by accounting for temporal directionality in the relationship between trust and communication. By examining differences between lagged and concurrent trust on interactions, these findings show that trust in an organization appears to catalyze information transmission to members of that organization.

Because communication ties follow increased trust, these results suggest that individuals seek relationships that require lower cognitive demands rather than establishing ties and waiting for those demands to fall over time as trust develops. In contexts of information exchange, relationships endowed with trust are more reliable, require less oversight, and have lower barriers for exchange (Cropanzano & Mitchell, 2005; Granovetter, 1985; McEvily et al., 2003; Uzzi, 1997). Establishing communication channels entails search costs and sustaining them requires regular effort (Cummings et al., 2006; Mariotti & Delbridge, 2012), and individuals appear to prefer to avoid the calculative processing associated with lower-trust relationships.

Effects of time on how individuals establish and utilize trust relationships have long remained unclear (Zaheer & Harris, 2006; Marks et al., 2001). This paper has demonstrated evidence that trust forecasts future communication, elucidating temporal directionality in the interrelationship observed between trust and communication in previous studies (De Jong et al., 2016). Overall, trust emerged more consistently as a *predictor* of communication in this setting, as opposed to an outcome. This has important implications for this paper’s setting, in which three coalition partners joined to form a larger organization for a brief period of time. This organization’s success hinged on effectively observing, analyzing, and responding to their operational environment, which required extensive information exchange within and across organizational sub-units. Organizational trust catalyzes information exchange; communication alone is not sufficient to build trust consistently, as the results demonstrated. In practice, such multifaceted organizations, especially those composed of relatively unfamiliar sub-units, will need to establish trust in order to facilitate information exchange. This is especially relevant for organizations operating in contexts characterized by extensive information exchange and disrupted task environments, where cognitive loads will be elevated. As demonstrated in the concurrent trust results, interactions were associated with positive, negative, and unclear effects on subsequent trust ratings, suggesting that communication cannot reliably foster trust. Future research can refine these results by examining how the *content* of interactions shapes or reflects trust relationships. Using natural language processing can isolate interaction networks characterized by types of team processes enumerated in Marks et al. (2001). Coupled with additional trust measures, such as interpersonal and competence-based trust, networks reflecting communication about transition processes (planning strategy, and strategy formulation), action processes (monitoring and coordination), and interpersonal processes (conflict management, affect management, and motivation) could provide additional insight into how specific types of communication shape, or are shaped by, specific types of trust.

Although we have taken steps in this paper to provide insight into trust dynamics that have heretofore received little attention, we acknowledge several limitations to our study. First, the type of trust examined here—individual’s reported trust in groups within an organization—pertains primarily to organizations composed of sets of interdependent roles that strive to achieve coordinated task execution in support of some larger organizational goal. As such, trust in the surveys was measured as confidence that members of a group will contribute to organizational success. This particular measure captures performance-oriented trust and may not generalize to more transactional trust that is critical for sustaining economic exchange relationships. Additionally, the concept of risk that underlies this type of trust relationship is not as immediate in this setting as it is in other contexts, particularly economic exchange (Mayer et al., 1995; Rousseau et al., 1998; Lewicki et al., 2006). As such, the results may not necessarily generalize to cases where risk is more immediately tangible, particularly at the individual level. Nevertheless, risk in this organization entails failing to achieve the organization’s goals and the repercussions of those failures, ranging in severity from taking on extra work if trustees are unable to complete their own tasks to sustaining casualties if a critical mistake is made during mission execution. Finally, trust was measured with a single survey item. Although the survey item captures the cognitive and behavioral foundations of trust from Lewis and Weigert (1985) and adheres to the Robinson (1996) definition of trust, a single item is inherently limited. A more comprehensive measure of trust would allow that measure to be distilled into distinct types of trust such as competence-based trust or interpersonal trust, providing greater insight into trust dynamics.

The timing of trust measures also impose some limitations. Trust was measured eight times over a narrow time period spanning about sixty working hours over ten days within the organization. Although this provides insight into initial organizational trust dynamics at narrow time scales, these results may not necessarily reflect how trust waxes or wanes over longer time scales covering multiple weeks, months, or years. As an example, the increasing standard deviations in individuals’ trust ratings over time demonstrate divergence in trust ratings over time. How long it may take for this divergence to revert to convergence, if at all, remains unclear. This underscores the argument made by Leenders et al. (2016) that a better understanding of dynamic group states and processes will help the field better understand when and for how long to measure key variables related to those states and processes. Additionally, more data points capturing trust would create opportunities for additional methods to evaluate trust over time, such as Granger causality models to examine forecasting between trust relationships and interaction. Such further analyses could provide a confirmatory complement to this paper’s analyses. Despite this paper’s limitations, these results have provided evidence to elucidate understanding of temporal and network-based perspectives of group states and processes.

## Conclusions

Trust research has grown substantially over the past three decades, yet much of what is known comes from static conceptualizations of trust. Further, many theoretical perspectives assume trust is shaped by interactions, without consideration of the impact of trust on future interactions. To advance an understanding of trust in organizations, this paper aimed to provide insight into the relationship between trust dynamics and communication dynamics as they evolve continuously over time. Findings show that trust forecasts communication structure and rates much more frequently than communication forecasts subsequent trust ratings or changes in trust. This manuscript provides initial evidence for path dependence in the relationship between group trust and communication, clarifying theories of relational exchange. These results show that establishing trust catalyzes information exchange, a critical aspect in the uncertain and uptempo settings in which military organizations often operate. In practice, these results emphasize that critical relationships between coalition partners need to be endowed with trust to facilitate information flow. Pre-emptively seeding those essential relationships with performance-oriented trust can ensure timely information flow during mission execution. These findings contribute to both general knowledge and practical application by providing a useful foundation for building the field’s limited understanding of trust dynamics (Rousseau et al., 1998; Brass et al., 2004; Zaheer & Harris, 2006). Many reviews of the trust literature have recognized the importance of observing the coevolution of interactions and trust, and they have advocated using network analyses to develop a better understanding of trust (Bromiley & Cummings, 1995; Mayer et al., 1995; Kramer, 1999; McEvily et al., 2003; Zaheer & Harris, 2006; Fulmer & Gelfand, 2012). This paper addresses those calls by demonstrating how static and dynamic analyses of communication networks provide unique insight into trust dynamics. As dynamic network methods (Snijders, 2001; Daraganova & Robins, 2013; Butts, 2008; Krivitsky & Handcock, 2014) continue to develop and enjoy increased usage, additional opportunities emerge to examine the mechanisms driving the evolution of group states and cognition over time.

## Open practices statement

Due to their sensitive nature, data and materials from the study are not publicly available. However, they may be made available from the authors upon reasonable request and with permission from the US Army Research Laboratory. None of the analyses in this study were preregistered.

## Data Availability

The data that support the findings of this study are available from the US Army Research Laboratory, but restrictions apply to the public availability of these data. The data may be made available from the authors upon reasonable request and with the permission of the US Army Research Laboratory.

## Appendix

In the remainder of this document, we provide full tables for our model results. We begin with our ERGM results for prior trust ($\tau _{t-1}$), current trust ($\tau_t$), and trust change ($\tau _{\Delta }$) in order before proceeding to STERGM results and, finally, relational event model results. ERGMs and STERGMs used identical model terms to control for other factors driving network structure, although the models occasionally had to omit a term to aid in model convergence. Likewise, the relational event models utilized identical terms across each time point and, to the best feasible extent, model terms with some analogue in the ERGM framework. Occasionally a relational event model omitted a term to enable the model to produce more stable results.

See Tables 10, 11, 12, 13, 14, 15, 16, 17, 18, 19, 20 and 21.

**Table 10:** ERGM, prior trust ($\tau _{t-1}$)

	Time 1		Time 2		Time 3		Time 4		Time 5		Time 6		Time 7		Time 8	
	Coef	SE	Coef	SE	Coef	SE	Coef	SE	Coef	SE	Coef	SE	Coef	SE	Coef	SE
Edges			$-$5.59***	0.40	$-$5.53***	0.34	$-$3.71***	0.48	$-$6.31***	0.40	$-$7.17***	0.67	$-$6.27***	0.51	$-$5.40***	0.53
UK			0.04	0.08	$-$0.07	0.07	$-$0.18	0.16	0.14*	0.07	0.54***	0.12	0.37***	0.11	$-$0.30*‘	0.14
US			$-$0.01	0.09	$-$0.00	0.09	0.28*	0.14	0.35***	0.07	0.40*	0.16	0.53***	0.09	$-$0.16	0.15
Same-cell ties			0.66***	0.13	0.77***	0.16	0.85***	0.21	0.62***	0.14	0.44*	0.20	1.13***	0.16	0.80***	0.20
Coordinative role in-ties			0.26’	0.15	$-$0.53**	0.16	0.48**	0.17	$-$0.34*	0.15	0.16	0.20	$-$0.62**	0.22	$-$0.11	0.19
Coordinative role out-ties			$-$0.12	0.15	0.62***	0.12	$-$1.83***	0.28	0.49***	0.14	$-$0.55*	0.22	0.76***	0.16	0.42**	0.15
Rank in-ties			$-$0.05	0.03	$-$0.06*	0.03	$-$0.09*	0.04	$-$0.00	0.03	0.03	0.05	0.05	0.04	$-$0.01	0.04
Rank out-ties			0.10**	0.03	0.10***	0.03	0.25***	0.04	0.14***	0.03	0.09’	0.04	0.09*	0.04	0.14***	0.04
SA in-ties			$-$0.51	0.53	0.53	0.45	1.33*	0.63	0.99*	0.47	1.26’	0.75	0.85	0.69	0.44	0.63
SA out-ties			0.35	0.55	0.93’	0.49	$-$3.66***	0.62	$-$1.18**	0.46	0.62	0.73	$-$0.90	0.65	$-$1.23*	0.57
Tie inertia			1.26***	0.10	0.94***	0.06	0.367***	0.02	0.503***	0.03	0.20***	0.01	0.11***	0.01	0.21***	0.01
Reciprocity			1.26***	0.22	0.73***	0.18	$-$0.08	0.28	1.99***	0.15	1.80***	0.24	1.92***	0.26	0.23	0.22
Transitivity			1.19***	0.08	0.80***	0.04	0.67***	0.05	1.30***	0.13	0.52***	0.06	0.87***	0.07	1.51***	0.13
Trust propensity in-ties			0.07*	0.03	0.02	0.03	$-$0.04	0.04	0.11***	0.03	0.06	0.05	0.04	0.04	$-$0.04	0.04
Trust propensity out-ties			$-$0.09*	0.05	$-$0.17***	0.04	$-$0.28***	0.06	$-$0.16***	0.04	$-$0.01	0.08	$-$0.25**	0.08	$-$0.26***	0.07
Prior trust ($\tau _{t-1})$			0.09*	0.05	0.09*	0.03	0.19***	0.06	0.07**	0.04	0.04	0.06	0.24***	0.07	0.25***	0.06
Null deviance			9435		9435		9435		9435		9435		9435		9435	
Residual deviance			1987		2194		1556		2895		1511		1458		1669	
Degrees of freedom			6790		6790		6790		6790		6790		6790		6790	

***p <.001,      **p < .01,     *p < .05

**Table 11:** ERGM, current trust ($\tau _{t}$)

	Time 1		Time 2		Time 3		Time 4		Time 5		Time 6		Time 7		Time 8	
	Coef	SE	Coef	SE	Coef	SE	Coef	SE	Coef	SE	Coef	SE	Coef	SE	Coef	SE
Edges	$-$5.60***	0.52	$-$5.35***	0.38	$-$5.32***	0.35	$-$3.76***	0.50	$-$6.19***	0.35	$-$7.14***	0.68	$-$6.41***	0.51	$-$5.51***	0.56
UK	$-$0.03	0.11	0.09	0.07	$-$0.04	0.07	$-$0.03	0.16	0.29***	0.06	0.56***	0.11	0.41***	0.08	$-$0.29*	0.13
US	0.04	0.11	0.02	0.08	$-$0.00	0.07	0.34*	0.14	0.41***	0.06	0.41**	0.15	0.56***	0.09	$-$0.13	0.14
Same-cell ties	1.48***	0.14	0.66***	0.14	0.79***	0.14	0.89***	0.21	0.74***	0.14	0.44*	0.22	1.13***	0.15	0.78***	0.20
Coordinative role in-ties	0.07	0.20	0.24’	0.15	$-$0.54**	0.15	0.51**	0.17	$-$0.40**	0.15	0.15	0.20	$-$0.61**	0.21	$-$0.17	0.19
Coordinative role out-ties	0.73***	0.17	$-$0.11	0.15	0.57***	0.11	$-$1.82***	0.28	0.32*	0.13	$-$0.54*	0.23	0.75***	0.16	0.52***	0.15
Rank in-ties	0.04	0.04	$-$0.05’	0.03	$-$0.07**	0.02	$-$0.10*	0.04	$-$0.03	0.03	0.03	0.05	0.04	0.04	0.01	0.03
Rank out-ties	0.16***	0.04	0.10**	0.03	0.11***	0.03	0.22***	0.04	0.11***	0.03	0.09*	0.04	0.08*	0.04	0.15***	0.04
SA in-ties	0.16	0.47	$-$0.47	0.51	0.61	0.43	1.34*	0.65	0.90*	0.45	1.20	0.75	0.88	0.64	0.48	0.63
SA out-ties	0.70	0.50	0.18	0.54	0.52	0.47	$-$3.22***	0.61	$-$0.95*	0.42	0.56	0.74	$-$1.00	0.62	$-$1.02’	0.58
Tie inertia			1.28***	0.10	0.94***	0.06	0.37***	0.02	0.46***	0.03	0.20***	0.01	0.11***	0.01	0.21***	0.01
Reciprocity	2.06***	0.27	1.23***	0.22	0.70***	0.17	$-$0.07	0.28	1.69***	0.15	1.81***	0.24	1.88***	0.26	0.26	0.22
Transitivity	1.05***	0.13	1.11***	0.07	0.81***	0.04	0.68***	0.05	1.01***	0.07	0.52***	0.06	0.90***	0.07	1.50***	0.13
Trust propensity in-ties	0.02	0.04	0.07*	0.03	0.02	0.03	$-$0.03	0.40	0.11***	0.03	0.06	0.04	0.05	0.04	$-$0.04	0.04
Trust propensity out-ties	$-$0.26***	0.06	0.02	0.04	$-$0.14***	0.04	$-$0.05	0.06	$-$0.09’	0.05	0.02	0.09	0.02	0.08	$-$0.29***	0.07
Current trust ($\tau _t$)	0.15*	0.06	$-$0.05	0.03	0.05	0.03	$-$0.05	0.05	0.04	0.03	0.01	0.07	$-$0.00	0.06	0.27***	0.05
Null deviance	9435		9435		9435		9435		9435		9435		9435		9435	
Residual deviance	1475		1982		2259		1572		2787		1512		1471		1659	
Degrees of freedom	6791		6790		6790		6790		6790		6790		6790		6790	

***p <.001,      **p < .01,     *p < .05

**Table 12:** ERGM, trust change ($\tau _{\Delta }$)

	Time 1		Time 2		Time 3		Time 4		Time 5		Time 6		Time 7		Time 8	
	Coef	SE	Coef	SE	Coef	SE	Coef	SE	Coef	SE	Coef	SE	Coef	SE	Coef	SE
Edges			$-$5.45***	0.33	$-$5.48***	0.40	$-$3.60***	0.52	$-$6.24***	0.35	$-$7.20***	0.69	$-$6.68***	0.52	$-$5.81***	0.57
UK			0.09	0.06	$-$0.06	0.08	$-$0.06	0.16	0.29***	0.06	0.55***	0.11	0.42***	0.09	$-$0.32*	0.13
US			0.02	0.08	$-$0.00	0.09	0.32*	0.14	0.42***	0.06	0.41*	0.16	0.58***	0.10	$-$0.18	0.15
Same-cell ties			0.67***	0.14	0.81***	0.17	0.91***	0.21	0.72***	0.14	0.43*	0.20	1.07***	0.16	0.68***	0.19
Coordinative role in-ties			0.25’	0.14	$-$0.53**	0.17	0.52**	0.18	$-$0.40**	0.14	0.14	0.19	$-$0.63**	0.22	$-$0.15	0.19
Coordinative role out-ties			$-$0.12	0.14	0.63***	0.12	$-$1.91***	0.29	0.72***	0.13	$-$0.56*	0.22	0.78***	0.16	0.62***	0.16
Rank in-ties			$-$0.05’	0.03	$-$0.06*	0.03	$-$0.09*	0.04	$-$0.03	0.03	0.03	0.05	0.04	0.04	0.00	0.04
Rank out-ties			0.10***	0.03	0.09**	0.03	0.25***	0.04	0.10***	0.03	0.08’	0.04	0.07’	0.04	0.14***	0.04
SA in-ties			$-$0.48	0.48	0.65	0.50	1.26’	0.65	0.88*	0.45	1.28’	0.74	1.00	0.62	0.50	0.64
SA out-ties			0.26	0.48	0.97’	0.51	$-$3.51***	0.64	$-$1.01*	0.43	0.62	0.71	$-$0.85	0.61	$-$1.14’	0.60
Tie inertia			1.26***	0.11	0.93***	0.06	0.37***	0.03	0.46***	0.03	0.20***	0.01	0.11***	0.01	0.22***	0.01
Reciprocity			1.21***	0.20	0.74***	0.19	$-$0.02	0.29	1.69***	0.16	1.81***	0.24	1.88***	0.26	0.30	0.22
Transitivity			1.11***	0.07	0.79***	0.04	0.67***	0.05	1.01***	0.07	0.52***	0.06	0.89***	0.07	1.60***	0.15
Trust propensity in-ties			0.06*	0.03	0.02	0.03	$-$0.04	0.04	0.11***	0.03	0.05	0.05	0.05	0.04	$-$0.03	0.04
Trust propensity out-ties			$-$0.01	0.03	$-$0.08**	0.03	$-$0.11**	0.04	$-$0.04	0.03	0.03	0.04	0.03	0.04	$-$0.03	0.03
Trust change ($\tau _{\Delta }$)			$-$0.06*	0.02	$-$0.06’	0.04	$-$0.13**	0.05	$-$0.02	0.03	$-$0.04	0.06	$-$0.17**	0.06	0.22***	0.08
Null deviance			9435		9435		9435		9435		9435		9435		9435	
Residual deviance			1977		2205		1559		2791		1513		1463		1717	
Degrees of freedom			6790		6790		6790		6790		6790		6790		6790	

***p <.001,      **p < .01,     *p < .05

**Table 13:** STERGM, formation: prior trust ($\tau _{t-1}$)

	Time 1-2		Time 2-3		Time 3-4		Time 4-5		Time 5-6		Time 6-7		Time 7-8	
	Coef	SE	Coef	SE	Coef	SE	Coef	SE	Coef	SE	Coef	SE	Coef	SE
Edges	$-$5.27***	0.47	$-$5.82***	0.48	$-$4.37***	0.82	$-$6.28***	0.44	$-$8.05***	1.31	$-$5.71***	0.84	$-$5.54***	0.68
UK	0.05	0.09	$-$0.17	0.11	$-$1.02***	0.23	0.47***	0.08	0.58**	0.18	$-$0.25	0.21	$-$0.60**	0.19
US	$-$0.11	0.12	$-$0.14	0.14	$-$0.13	0.19	0.51***	0.08	$-$0.77*	0.34	0.22	0.18	$-$0.36*	0.18
Same-cell ties	0.69***	0.18	0.87***	0.20	0.96***	0.27	0.66***	0.19	0.62’	0.34	1.37***	0.24	0.70*	0.28
Coordinative role in-ties	0.07	0.16	$-$0.63**	0.20	0.20	0.25	$-$0.42*	0.17	$-$0.15	0.33	$-$0.15	0.30	$-$0.16	0.23
Coordinative role out-ties	$-$0.11	0.16	0.52***	0.14	$-$0.88*	0.44	0.26’	0.13	$-$0.06	0.35	0.50*	0.24	0.35*	0.17
Rank in-ties	$-$0.05	0.04	$-$0.06’	0.03	$-$0.05	0.05	$-$0.06’	0.03	$-$0.07	0.08	0.09	0.06	$-$0.05	0.04
Rank out-ties	0.07’	0.04	0.13***	0.04	0.40***	0.06	0.12***	0.03	$-$0.04	0.07	0.17**	0.06	0.13**	0.05
SA in-ties	$-$0.36	0.61	0.68	0.59	1.65’	0.92	1.04*	0.50	2.47’	1.34	$-$0.40	0.91	1.22	0.75
SA out-ties	0.43	0.59	1.75**	0.62	$-$5.43***	0.85	$-$1.394**	0.46	2.66*	1.35	$-$2.26*	0.89	$-$0.61	0.72
Inertia			0.85***	0.18	0.77***	0.10	0.52***	0.04	0.27***	0.05	0.23***	0.02	0.17***	0.02
Reciprocity	1.10***	0.22	0.62**	0.19	0.58*	0.26	1.46***	0.17	2.08***	0.27	1.10***	0.27	$-$0.62*	0.27
Transitivity	0.68***	0.04	0.52***	0.03	0.93***	0.15	0.65***	0.04	0.77***	0.20	0.98***	0.15	0.82***	0.06
Trust propensity in-ties	0.07’	0.04	0.02	0.04	0.05	0.06	0.12***	0.03	0.16*	0.08	$-$0.03	0.06	$-$0.06	0.04
Trust propensity out-ties	$-$0.14**	0.05	$-$0.28***	0.06	$-$0.49***	0.09	$-$0.04	0.05	$-$0.01	0.12	$-$0.19	0.13	$-$0.29**	0.09
Prior trust ($\tau _{t-1}$)	0.12*	0.05	0.15***	0.05	0.29***	0.07	0.01	0.04	$-$0.10	0.09	0.21*	0.10	0.30***	0.07
Null deviance	9435		9435		9435		9435		9435		9435		9435	
Residual deviance	1952		2122		1582		2688		1390		1345		1560	
Degrees of freedom	6775		6774		6774		6774		6774		6774		6774	

***p <.001,      **p < .01,     *p < .05

**Table 14:** STERGM, persistence: prior trust ($\tau _{t-1}$)

	Time 1-2		Time 2-3		Time 3-4		Time 4-5		Time 5-6		Time 6-7		Time 7-8	
	Coef	SE	Coef	SE	Coef	SE	Coef	SE	Coef	SE	Coef	SE	Coef	SE
Edges	$-$1.45	1.52	$-$1.86	1.35	2.06’	1.10	$-$3.10*	1.38	$-$4.40***	1.02	$-$2.07	1.35	0.22	1.48
UK	$-$0.16	0.37	$-$0.47’	0.26	$-$0.14	0.3	0.27	0.43	0.02	0.21	0.64*	0.29	$-$0.48	0.35
US	$-$0.22	0.35	$-$0.17	0.28	0.32	0.28	0.32	0.31	0.35’	0.21	0.42	0.34	$-$1.18**	0.39
Same-cell ties	0.48	0.41	0.31	0.33	0.02	0.29	0.82’	0.42	0.10	0.24	0.85**	0.32	0.28	0.42
Coordinative role in-ties	0.47	0.43	0.08	0.32	0.37	0.29	0.35	0.39	0.13	0.26	$-$0.29	0.37	0.38	0.45
Coordinative role out-ties	$-$0.03	0.39	0.80*	0.39	$-$1.97***	0.31	0.09	0.55	$-$0.82**	0.25	1.30***	0.39	0.30	0.38
Rank in-ties	$-$0.14	0.11	0.08	0.09	$-$0.04	0.08	0.25**	0.09	0.09	0.07	0.06	0.09	0.03	0.11
Rank out-ties	0.33*	0.13	0.25**	0.10	0.10	0.09	$-$0.04	0.11	0.13’	0.07	$-$0.01	0.10	0.25*	0.12
SA in-ties	$-$3.00’	1.62	0.67	1.27	$-$0.24	1.12	$-$0.25	1.60	0.73	1.01	$-$0.51	1.45	$-$2.94’	1.74
SA out-ties	$-$0.50	1.94	0.91	1.37	$-$4.62***	1.28	$-$2.77*	1.39	$-$0.23	1.04	$-$1.65	01.33	$-$2.70	1.83
Inertia	0.67***	0.18	0.41***	0.09	0.33***	0.03	0.26***	0.04	0.14***	0.01	0.04***	0.01	0.11***	0.02
Reciprocity	1.10*	0.43	$-$0.56’	0.34	0.31	0.32	1.23**	0.43	1.06***	0.26	1.76***	0.35	0.57	0.41
Transitivity	0.08	0.21	0.32’	0.17	0.31*	0.14	0.19	0.22	0.18	0.13	0.49**	0.18	0.09	0.22
Trust propensity in-ties	0.05	0.10	$-$0.13’	0.08	$-$0.23***	0.07	0.03	0.09	$-$0.03	0.06	0.01	0.08	$-$0.02	0.09
Trust propensity out-ties	$-$0.21	0.17	$-$0.19’	0.11	$-$0.27**	0.10	$-$0.13	0.16	$-$0.13	0.13	$-$0.27	0.20	$-$0.38’	0.22
Prior trust ($\tau _{t-1}$)	0.20	0.17	0.12*	0.06	0.15’	0.09	0.33*	0.13	0.19*	0.09	0.32*	0.16	0.36*	0.16
Null deviance	9435		9435		9435		9435		9435		9435		9435	
Residual deviance	1952		2122		1582		2688		1390		1345		1560	
Degrees of freedom	6775		6774		6774		6774		6774		6774		6774	

***p <.001,      **p < .01,     *p < .05

**Table 15:** STERGM, formation: current trust ($\tau _{t}$)

	Time 1-2		Time 2-3		Time 3-4		Time 4-5		Time 5-6		Time 6-7		Time 7-8	
	Coef	SE	Coef	SE	Coef	SE	Coef	SE	Coef	SE	Coef	SE	Coef	SE
Edges	$-$5.03***	0.37	$-$5.68***	0.49	$-$4.49***	0.82	$-$6.24***	0.43	$-$8.14***	1.31	$-$5.70***	0.88	$-$5.84***	0.73
UK	0.11	0.07	$-$0.16	0.11	$-$0.90***	0.24	0.47***	0.07	0.56**	0.18	$-$0.24	0.21	$-$0.58**	0.19
US	$-$0.09	0.09	$-$0.11	0.14	$-$0.05	0.19	0.52***	0.07	$-$0.80*	0.34	0.22	0.18	$-$0.30’	0.18
Same$-$cell ties	0.67***	0.17	0.90***	0.20	1.06***	0.26	0.67***	0.18	0.61’	0.34	1.34***	0.24	0.64*	0.27
Coordinative role in-ties	0.05	0.15	$-$0.62**	0.20	0.21	0.25	$-$0.41*	0.17	$-$0.12	0.33	$-$0.12	0.30	$-$0.20	0.23
Coordinative role out-ties	$-$0.06	0.14	0.51***	0.14	$-$0.91*	0.43	0.26’	0.13	$-$0.04	0.35	0.48*	0.23	0.48**	0.17
Rank in-ties	$-$0.06’	0.03	$-$0.06’	0.03	$-$0.07	0.05	$-$0.06’	0.03	$-$0.07	0.08	0.08	0.06	$-$0.05	0.04
Rank out-ties	0.06’	0.03	0.13**	0.04	0.37***	0.06	0.12***	0.03	$-$0.03	0.07	0.16**	0.06	0.15**	0.06
SA in-ties	$-$0.34	0.55	0.59	0.60	1.74’	0.92	0.97*	0.48	2.45’	1.33	$-$0.48	0.93	1.25’	0.75
SA out-ties	0.12	0.52	1.46*	0.63	$-$4.99***	0.81	$-$1.39**	0.47	2.37’	1.36	$-$2.32**	0.87	$-$0.23	0.77
Inertia			0.88***	0.18	0.73***	0.10	0.52***	0.04	0.27***	0.05	0.23***	0.02	0.18***	0.02
Reciprocity	1.07***	0.19	0.59**	0.20	0.56*	0.26	1.48***	0.16	2.09***	0.28	1.08***	0.26	$-$0.61*	0.27
Transitivity	0.70***	0.04	0.53***	0.03	0.96***	0.15	0.64***	0.04	0.76***	0.2	0.99***	0.15	0.81***	0.06
Trust propensity in-ties	0.08*	0.03	0.02	0.04	0.05	0.06	0.11**	0.03	0.16	0.08	$-$0.03	0.06	$-$0.06	0.04
Trust propensity out-ties	0.05	0.04	$-$0.17**	0.05	$-$0.12	0.08	$-$0.02	0.05	0.12	0.16	$-$0.02	0.11	$-$0.32***	0.08
Current trust ($\tau _{t}$)	$-$0.10***	0.02	0.05	0.04	$-$0.07	0.06	$-$0.01	0.04	$-$0.21’	0.12	0.05	0.08	0.31***	0.06
Null deviance	9435		9435		9435		9435		9435		9435		9435	
Residual deviance	1949		2132		1591		2695		1386		1352		1550	
Degrees of freedom	6775		6774		6774		6774		6774		6774		6774	

***p <.001,      **p < .01,     *p < .05

**Table 16:** STERGM, persistence: current trust ($\tau _{t}$)

	Time 1-2		Time 2-3		Time 3-4		Time 4-5		Time 5-6		Time 6-7		Time 7-8	
	Coef	SE	Coef	SE	Coef	SE	Coef	SE	Coef	SE	Coef	SE	Coef	SE
Edges	$-$1.69	1.52	$-$1.75	1.37	2.40*	1.10	$-$3.11*	1.40	$-$4.43***	1.03	$-$2.66*	1.32	0.18	1.46
UK	$-$0.09	0.37	$-$0.53*	0.26	0.01	0.31	0.30	0.42	0.05	0.21	0.60*	0.29	$-$0.45	0.35
US	$-$0.12	0.33	$-$0.16	0.27	0.42	0.28	0.40	0.31	0.37’	0.21	0.57’	0.32	$-$1.15**	0.38
Same-cell ties	0.48	0.41	0.28	0.34	0.02	0.30	0.73’	0.40	0.11	0.24	0.95**	0.32	0.28	0.41
Coordinative role in-ties	0.48	0.43	0.10	0.33	0.33	0.29	0.41	0.39	0.16	0.26	$-$0.36	0.37	0.26	0.44
Coordinative role out-ties	0.06	0.40	0.77*	0.38	$-$2.07***	0.33	$-$0.14	0.56	$-$0.77	0.26	1.41***	0.40	0.39	0.38
Rank in-ties	$-$0.14	0.11	0.08	0.09	$-$0.04	0.08	0.29**	0.10	0.09	0.07	0.06	0.09	0.02	0.11
Rank out-ties	0.38**	0.13	0.27	0.10	0.07	0.09	0.02	0.11	0.13’	0.07	$-$0.00	0.10	0.26*	0.12
SA in-ties	$-$2.99’	1.57	0.61	1.29	$-$0.33	1.13	$-$0.90	1.61	0.67	1.00	$-$0.26	1.45	$-$2.93’	1.77
SA out-ties	$-$0.13	1.98	0.65	1.42	$-$4.35***	1.30	$-$1.33	1.41	$-$0.41	1.05	$-$1.65	1.35	$-$2.59	1.86
Inertia	0.72***	0.18	0.41***	0.09	0.34***	0.03	0.25***	0.04	0.14***	0.01	0.04***	0.01	0.12***	0.02
Reciprocity	1.16**	0.43	$-$0.60’	0.34	0.25	0.33	1.00*	0.42	1.05***	0.26	1.76***	0.36	0.58	0.40
Transitivity	0.11	0.20	0.35*	0.18	0.33*	0.15	0.12	0.22	0.19	0.13	0.51**	0.18	0.10	0.21
Trust propensity in-ties	0.05	0.10	$-$0.13’	0.08	$-$0.23***	0.07	$-$0.01	0.09	$-$0.03	0.06	0.01	0.08	$-$0.02	0.09
Trust propensity out-ties	$-$0.18	0.15	$-$0.23*	0.11	0.09	0.13	$-$0.10	0.20	$-$0.05	0.14	0.09	0.19	$-$0.34	0.21
Current trust ($\tau _{t}$)	0.13	0.13	0.16*	0.06	$-$0.25*	0.12	0.24	0.14	0.13	0.11	0.01	0.13	0.31*	0.14
Null deviance	9435		9435		9435		9435		9435		9435		9435	
Residual deviance	1949		2132		1591		2695		1386		1352		1550	
Degrees of freedom	6775		6774		6774		6774		6774		6774		6774	

***p <.001,      **p < .01,     *p < .05

**Table 17:** STERGM, formation: trust change ($\tau _{\Delta }$)

	Time 1-2		Time 2-3		Time 3-4		Time 4-5		Time 5-6		Time 6-7		Time 7-8	
	Coef	SE	Coef	SE	Coef	SE	Coef	SE	Coef	SE	Coef	SE	Coef	SE
Edges	$-$5.08***	0.46	$-$5.93***	0.48	$-$4.34***	0.84	$-$6.23***	0.41	$-$7.97***	1.28	$-$6.01***	0.87	$-$6.34***	0.72
UK	0.09	0.09	$-$0.16	0.12	$-$0.94***	0.23	0.47***	0.06	0.54**	0.18	$-$0.2	0.21	$-$0.50**	0.19
US	$-$0.10	0.12	$-$0.12	0.13	$-$0.06	0.20	0.50***	0.07	$-$0.80*	0.34	0.27	0.18	$-$0.23	0.18
Same-cell ties	0.70***	0.18	0.89***	0.19	1.01***	0.28	0.67***	0.18	0.59’	0.35	1.31***	0.24	0.52*	0.25
Coordinative role in-ties	0.07	0.16	$-$0.57**	0.20	0.19	0.26	$-$0.41*	0.17	$-$0.13	0.32	$-$0.16	0.29	$-$0.23	0.24
Coordinative role out-ties	$-$0.09	0.16	0.54***	0.14	$-$1.10*	0.43	0.25*	0.13	$-$0.06	0.35	0.54	0.24	0.50**	0.18
Rank in-ties	$-$0.06	0.04	$-$0.06’	0.03	$-$0.05	0.05	$-$0.06*	0.03	$-$0.07	0.08	0.08	0.06	$-$0.06	0.04
Rank out-ties	0.06’	0.03	0.11**	0.04	0.40***	0.06	0.12***	0.03	$-$0.02	0.07	0.15	0.06	0.12*	0.05
SA in-ties	$-$0.34	0.60	0.57	0.61	1.62’	0.91	0.98*	0.47	2.42’	1.33	$-$0.24	0.95	1.35’	0.75
SA out-ties	0.22	0.58	2.05**	0.67	$-$5.38***	0.81	$-$1.40**	0.46	2.56’	1.31	$-$2.25	0.89	$-$0.17	0.73
Inertia			0.88***	0.17	0.81***	0.10	0.52***	0.03	0.27***	0.05	0.24	0.02	0.18***	0.02
Reciprocity	1.08***	0.22	0.62**	0.20	0.54*	0.26	1.47***	0.16	2.07***	0.27	1.10	0.25	$-$0.62*	0.25
Transitivity	0.69***	0.04	0.53***	0.03	0.99***	0.15	0.64***	0.04	0.78***	0.20	0.96	0.15	0.82***	0.06
Trust propensity in-ties	0.07’	0.04	0.02	0.03	0.04	0.06	0.11***	0.03	0.16*	0.08	$-$0.02	0.06	$-$0.05	0.04
Trust propensity out-ties	$-$0.03	0.03	$-$0.11***	0.03	$-$0.20***	0.05	$-$0.03	0.03	$-$0.11	0.07	0.05	0.06	0.05	0.04
Trust change ($\tau _{\Delta }$)	$-$0.08***	0.02	$-$0.13**	0.05	$-$0.30***	0.07	$-$0.01	0.03	$-$0.03	0.10	$-$0.10	0.09	0.26**	0.10
Null deviance	9435		9435		9435		9435		9435		9435		9435	
Residual deviance	1947		2130		1573		2698		1397		1351		1578	
Degrees of freedom	6775		6774		6774		6774		6774		6774		6774	

***p <.001,      **p < .01,     *p < .05

**Table 18:** STERGM, persistence: trust change ($\tau _{\Delta }$)

	Time 1-2		Time 2-3		Time 3-4		Time 4-5		Time 5-6		Time 6-7		Time 7-8	
	Coef	SE	Coef	SE	Coef	SE	Coef	SE	Coef	SE	Coef	SE	Coef	SE
Edges	$-$1.47	1.52	$-$1.55	1.37	2.39*	1.11	$-$3.12*	1.35	$-$4.55***	1.04	$-$2.73*	1.31	$-$0.45	1.41
UK	$-$0.11	0.36	$-$0.48’	0.25	$-$0.08	0.30	0.36	0.42	0.06	0.22	0.64*	0.28	$-$0.48	0.37
US	$-$0.10	0.33	$-$0.10	0.28	0.32	0.28	0.46	0.31	0.41’	0.21	0.51	0.32	$-$1.14**	0.39
Same-cell ties	0.42	0.40	0.33	0.34	0.03	0.30	0.75’	0.40	0.10	0.25	0.87**	0.33	0.20	0.39
Coordinative role in-ties	0.46	0.43	0.12	0.32	0.37	0.29	0.39	0.39	0.13	0.26	$-$0.35	0.38	0.25	0.45
Coordinative role out-ties	$-$0.03	0.40	0.89*	0.37	$-$2.03***	0.32	0.03	0.55	$-$0.76**	0.25	1.34***	0.39	0.41	0.38
Rank in-ties	$-$0.15	0.11	0.06	0.08	$-$0.04	0.07	0.26**	0.09	0.08	0.07	0.04	0.09	$-$0.01	0.11
Rank out-ties	0.37**	0.13	0.23*	0.09	0.09	0.09	$-$0.05	0.11	0.11	0.07	$-$0.04	0.10	0.20	0.12
SA in-ties	$-$2.92’	1.55	0.88	1.24	$-$0.27	1.11	$-$0.56	1.57	0.82	1.02	$-$0.40	1.45	$-$2.66	1.71
SA out-ties	$-$0.52	1.96	0.55	1.38	$-$4.63***	1.27	$-$2.38’	1.40	$-$0.37	1.04	$-$1.29	1.34	$-$2.54	0.19
Inertia	0.71***	0.18	0.42***	0.09	0.34***	0.03	0.26***	0.04	0.14***	0.01	0.05***	0.01	0.12***	0.02
Reciprocity	1.14**	0.43	$-$0.58’	0.34	0.29	0.33	1.12**	0.42	1.07***	0.27	1.69***	0.35	0.43	0.39
Transitivity	0.11	0.21	0.33’	0.18	0.30*	0.15	0.14	0.22	0.19	0.13	0.55**	0.19	0.19	0.21
Trust propensity in-ties	0.05	0.10	$-$0.13’	0.08	$-$0.23***	0.07	0.01	0.09	$-$0.02	0.06	0.03	0.08	0.01	0.09
Trust propensity out-ties	$-$0.05	0.09	$-$0.08	0.09	$-$0.15*	0.06	0.22*	0.10	0.10’	0.06	0.12	0.09	0.07	0.09
Trust change ($\tau _{\Delta }$)	0.01	0.11	0.21	0.17	$-$0.18*	0.08	$-$0.08	0.08	$-$0.12	0.11	$-$0.21’	0.13	0.12	0.27
Null deviance	9435		9435		9435		9435		9435		9435		9435	
Residual deviance	1947		2130		1573		2698		1397		1351		1578	
Degrees of freedom	6775		6774		6774		6774		6774		6774		6774	

***p <.001,      **p < .01,     *p < .05

**Table 19:** Relational Event Model, prior trust ($\tau _{t-1}$)

	Time 1		Time 2		Time 3		Time 4		Time 5		Time 6		Time 7		Time 8	
	Coef	SE	Coef	SE	Coef	SE	Coef	SE	Coef	SE	Coef	SE	Coef	SE	Coef	SE
Baseline sending			$-$5.13***	0.19	$-$8.04***	0.21	$-$8.51***	0.31	$-$8.49***	0.19	$-$10.13***	0.40	$-$8.41***	0.43	$-$9.05***	0.33
Coordinative role receiving			0.44***	0.05	0.04	0.06	0.25**	0.08	0.15**	0.05	0.28**	0.10	0.24*	0.11	0.29***	0.08
Coordinative role sending			0.85***	0.06	0.46***	0.05	0.06	0.10	0.27***	0.05	0.31**	0.10	0.15	0.11	0.15*	0.07
Rank receiving			0.06**	0.01	0.15***	0.01	0.11***	0.02	0.07***	0.01	0.18***	0.02	0.16***	0.03	0.14***	0.02
Rank sending			$-$0.04**	0.01	0.19***	0.02	0.31***	0.02	0.20***	0.01	0.20***	0.02	0.28***	0.03	0.25***	0.02
SA receiving			$-$0.97***	0.24	$-$0.55**	0.19	$-$0.45’	0.27	0.06	0.18	1.23**	0.40	$-$0.08	0.48	$-$0.47	0.30
SA sending			$-$3.19***	0.35	0.97***	0.22	$-$0.66*	0.28	0.65***	0.17	$-$0.60	0.42	$-$2.15***	0.48	$-$1.05***	0.31
Indegree receiving			1.26*	0.54	2.92***	0.75	4.96***	0.60	4.76***	0.40	5.10**	1.79			6.48***	0.60
Outdegree sending			4.97***	0.14	3.37***	0.19	1.62***	0.23	2.17***	0.20	2.43***	0.53	3.55***	0.51	1.54***	0.39
Recency receiving			2.28***	0.06	0.81***	0.08	1.13***	0.10	1.70***	0.06	2.03***	0.12	2.53***	0.13	1.71***	0.10
Recency sending			4.09***	0.07	3.81***	0.08	4.01***	0.11	3.32***	0.07	2.66***	0.15	2.87***	0.15	3.38***	0.11
Transitivity			0.34***	0.01	0.05***	0.00	0.04***	0.00	0.04***	0.00	0.14***	0.01	0.17***	0.02	0.07***	0.01
Cyclicity			$-$0.30***	0.01	$-$0.01***	0.00	$-$0.01’	0.01	$-$0.01***	0.00	$-$0.08***	0.02	0.00	0.02	$-$0.02**	0.01
AB-BA			$-$1.01***	0.15	$-$0.21	0.29	0.40	0.37	0.77***	0.18	2.50***	0.37	0.80***	0.28	0.62’	0.35
AB-BY			$-$1.24***	0.14	$-$0.95***	0.21	$-$0.61*	0.28	$-$0.54*	0.21			1.77***	0.19	$-$0.32	0.33
AB-XY			$-$3.83***	0.07	$-$2.05***	0.08	$-$1.67***	0.12	$-$1.29***	0.09	0.23	0.27	$-$2.02***	0.16	$-$1.20***	0.14
AB-AY			1.61***	0.06	3.71***	0.08	4.01***	0.12	4.14***	0.09	5.29***	0.27	3.07***	0.16	4.15***	0.14
Avg. trust receiving			0.06***	0.01	$-$0.10***	0.01	$-$0.13***	0.02	$-$0.08***	0.01	$-$0.12***	0.02	$-$0.01	0.03	$-$0.08***	0.02
Avg. trust sending			$-$3.20***	0.02	$-$0.16***	0.02	$-$0.12***	0.03	$-$0.31***	0.02	$-$0.25***	0.05	$-$0.49***	0.06	$-$0.29***	0.04
Prior trust ($\tau _{t-1}$)			$-$0.24***	0.02	0.05**	0.02	0.01	0.03	0.13***	0.02	0.07	0.04	0.27***	0.05	0.19***	0.03
Null deviance			17554		42448		23753		57221		14611		13220		23011	
Residual deviance			13006		20148		12679		33263		9633		8986		13447	
Degrees of freedom			1001		2702		1385		3272		811		707		1307	

***p <.001,      **p < .01,     *p < .05

**Table 20:** Relational Event Model, current trust ($\tau _{t}$)

	Time 1		Time 2		Time 3		Time 4		Time 5		Time 6		Time 7		Time 8	
	Coef	SE	Coef	SE	Coef	SE	Coef	SE	Coef	SE	Coef	SE	Coef	SE	Coef	SE
Baseline sending	$-$13.42***	0.50	$-$7.18***	0.25	$-$8.36***	0.23	$-$8.54***	0.32	$-$0.99***	0.11	$-$8.89***	0.39	$-$8.60***	0.38	$-$9.05***	0.33
Coordinative role receiving	1.22***	0.08	0.74***	0.06	0.07	0.06	0.26***	0.08	0.59***	0.04	0.21*	0.10	0.36***	0.09	0.32***	0.08
Coordinative role sending	1.37***	0.11	0.03	0.07	0.49***	0.05	0.06	0.10	0.31***	0.04	0.18’	0.10	$-$0.05	0.10	0.21**	0.07
Rank receiving	0.18***	0.02	0.12***	0.01	0.11***	0.01	0.11***	0.02	$-$0.14***	0.01	0.13***	0.02	0.08***	0.02	0.14***	0.02
Rank sending	1.05***	0.04	0.28***	0.02	0.18***	0.02	0.31***	0.02	0.00	0.01	0.19***	0.02	0.18***	0.03	0.26***	0.02
SA receiving	2.80***	0.38	$-$2.33***	0.24	$-$0.04	0.20	$-$0.42	0.27	$-$1.23***	0.13	0.21	0.38	0.26	0.39	$-$0.62*	0.29
SA sending	$-$0.82*	0.37	$-$2.97***	0.23	0.68**	0.24	$-$0.62*	0.27	$-$4.07***	0.12	0.24	0.43	$-$1.25**	0.41	$-$0.94**	0.32
Indegree receiving	$-$8.48***	2.02	2.08	1.36	4.66***	0.58	5.21***	0.56	8.71***	0.09	5.53***	0.89	4.23***	0.95	5.97***	0.68
Outdegree sending	$-$3.88***	0.70	3.16***	0.20	2.83***	0.23	1.47***	0.24	$-$1.50***	0.22	3.28***	0.44	2.97***	0.48	1.77***	0.38
Recency receiving	1.01***	0.09	1.31***	0.08	0.91***	0.08	1.15***	0.10	1.70***	0.05	1.93***	0.12	1.69***	0.12	1.72***	0.10
Recency sending	3.90***	0.09	4.19***	0.09	3.88***	0.08	3.97***	0.11	3.25***	0.06	2.68***	0.14	2.99***	0.13	3.38***	0.11
Transitivity	0.83***	0.02	0.13***	0.01	0.05***	0.00	0.04***	0.00	0.06***	0.00	0.14***	0.01	0.23***	0.02	0.07***	0.01
Cyclicity	0.52***	0.03	0.02*	0.01	$-$0.01***	0.00	$-$0.01’	0.01	$-$0.03***	0.00	$-$0.03’	0.02	0.00	0.02	$-$0.02***	0.01
AB-BA	0.47**	0.17	1.30***	0.18	$-$0.32	0.33	0.45	0.36			1.25***	0.34	0.10	0.55	0.44	0.37
AB-BY	$-$3.15***	0.42	$-$0.82**	0.26	$-$1.19***	0.27	$-$0.79*	0.31	$-$3.60***	0.54	$-$0.51	0.45	1.27***	0.25	$-$0.31	0.32
AB-XY	$-$4.53***	0.10	$-$1.73***	0.10	$-$1.94***	0.09	$-$1.68***	0.12	$-$2.38***	0.06	$-$0.78***	0.18	$-$0.78***	0.18	$-$1.26***	0.14
AB-AY	1.09***	0.08	3.83***	0.10	3.96***	0.09	4.01***	0.12	3.68***	0.06	4.10***	0.18	3.93***	0.18	4.09***	0.14
Avg. trust receiving	$-$0.08***	0.02	$-$0.05**	0.01	$-$0.06***	0.01	$-$0.13***	0.02	$-$0.29***	0.01	$-$0.12***	0.02	$-$0.05*	0.02	$-$0.08***	0.02
Avg. trust sending	$-$0.35***	0.03	$-$0.16***	0.03	$-$0.11***	0.02	$-$0.08**	0.03	$-$0.40***	0.01	$-$0.07	0.05	$-$0.20***	0.05	$-$0.31***	0.04
current trust ($\tau _{t}$)	0.05	0.03	0.10***	0.02	$-$0.01	0.02	$-$0.03	0.03	0.15***	0.01	$-$0.08*	0.04	0.06	0.04	0.21***	0.03
Null deviance	8434		17554		42448		23753		57221		14611		13220		23011	
Residual deviance	7745		11179		20060		12676		39028		9096		8645		13431	
Degrees of freedom	399		1001		2702		1385		3273		810		706		1307	

***p <.001,      **p < .01,     *p < .05

**Table 21:** Relational Event Model, trust change ($\tau _{\Delta }$)

	Time 1		Time 2		Time 3		Time 4		Time 5		Time 6		Time 7		Time 8	
	Coef	SE	Coef	SE	Coef	SE	Coef	SE	Coef	SE	Coef	SE	Coef	SE	Coef	SE
Baseline sending			$-$6.30***	0.26	$-$8.41***	0.23	$-$7.37***	0.29	$-$9.01***	0.20	$-$9.38***	0.41	$-$9.10***	0.41	$-$9.87***	0.31
Coordinative role receiving			1.53***	0.06	0.06	0.06	0.13’	0.08	0.11*	0.05	0.30**	0.10	0.21*	0.10	0.46***	0.07
Coordinative role sending			$-$0.22**	0.08	0.50***	0.05	0.18’	0.10	0.23***	0.05	0.14	0.10	0.03	0.10	0.05	0.07
Rank receiving			0.14***	0.02	0.11***	0.01	0.03’	0.02	0.06***	0.01	0.15***	0.02	0.08***	0.02	0.13***	0.02
Rank sending			0.22***	0.02	0.18***	0.02	0.21***	0.02	0.20***	0.01	0.19***	0.03	0.17***	0.03	0.27***	0.02
SA receiving			$-$1.82***	0.26	0.13	0.21	1.14***	0.27	0.12	0.18	0.05	0.39	$-$0.06	0.41	$-$1.00***	0.26
SA sending			0.10	0.25	0.89***	0.25	$-$1.69***	0.25	0.78***	0.17	0.14	0.44	$-$0.94*	0.43	$-$0.27	0.29
Indegree receiving			4.25	2.23	4.35***	0.67	2.73**	0.91	4.38***	0.49	5.68***	1.05	5.40***	0.99	6.91***	0.47
Outdegree sending			$-$2.08**	0.63	2.71***	0.23	2.83***	0.19	2.10**	0.21	2.64***	0.52	1.66*	0.66	2.13***	0.31
Recency receiving			1.58***	0.08	0.94***	0.08	1.32***	0.10	1.64***	0.06	1.94***	0.12	1.73***	0.13	1.67***	0.09
Recency sending			3.99***	0.10	3.89***	0.08	4.17***	0.11	3.41***	0.07	2.79***	0.15	3.19***	0.14	3.53***	0.10
Transitivity			0.18***	0.01	0.05***	0.00	0.05***	0.00	0.04***	0.00	0.14***	0.01	0.21***	0.02	0.07***	0.01
Cyclicity			$-$0.22***	0.02	$-$0.01***	0.00	$-$0.01’	0.01	$-$0.01**	0.00	$-$0.06***	0.02	0.03	0.02	$-$0.05***	0.01
AB-BA			2.65***	0.19	$-$0.08	0.29	$-$0.39	0.63	0.51*	0.22	1.52***	0.36	0.99*	0.45	2.70***	0.18
AB-BY					$-$1.21***	0.27			$-$0.67**	0.25	0.13	0.42	$-$0.41	0.56		
AB-XY			$-$0.73***	0.13	$-$1.95***	0.09	$-$0.97***	0.13	$-$1.06***	0.10	$-$0.50*	0.21	$-$0.47*	0.22	$-$0.96***	0.14
AB-AY			4.84***	0.13	3.95***	0.09	4.54***	0.13	4.35***	0.10	4.44***	0.21	4.42***	0.22	4.63***	0.14
Avg. trust receiving			$-$0.15***	0.02	$-$0.07***	0.01	$-$0.23***	0.02	$-$0.07***	0.01	$-$0.11***	0.02	$-$0.05*	0.02	$-$0.07***	0.02
Avg. trust sending			$-$0.32***	0.02	$-$0.12***	0.01	$-$0.11***	0.02	$-$0.17***	0.01	$-$0.14***	0.02	$-$0.13***	0.02	$-$0.06***	0.02
Trust change ($\tau _{\Delta }$)			0.05***	0.01	$-$0.06**	0.02	$-$0.08**	0.03	$-$0.10***	0.01	$-$0.07	0.04	$-$0.04	0.04	0.70***	0.03
Null deviance			17554		42448		23753		57221		14611		13220		23011	
Residual deviance			11808		20056		12883		33272		9086		8598		13671	
Degrees of freedom			1002		2702		1386		3272		810		706		1308	

***p <.001,      **p < .01,     *p < .05

## Abbreviations

ERGM: Exponential-family random graph model
STERGM: Separable temporal exponential-family random graph model
SA: Situation awareness
